# Immunotherapy and postoperative bone defect repair strategies based on osteosarcoma tumor microenvironment characteristics: balancing antitumor effects and promotion of bone regeneration

**DOI:** 10.1093/rb/rbag031

**Published:** 2026-03-05

**Authors:** Qiang Guo, Chunxiao Ran, Zixin Wang, Xiuzhi Zhang, Yiqun Yao, Xiaoyan Chen, Yan Zhang, Jiahui Yang, Yuhe You, Dewei Zhao

**Affiliations:** Department of Orthopedics, Affiliated Zhongshan Hospital of Dalian University, Dalian, Liaoning 116001, China; Zhongshan Clinical College, Dalian University, Dalian, Liaoning 116001, China; Orthopaedic Medical Research Center , Dalian University, Dalian, Liaoning 116001, China; Department of Orthopedics, Affiliated Zhongshan Hospital of Dalian University, Dalian, Liaoning 116001, China; Zhongshan Clinical College, Dalian University, Dalian, Liaoning 116001, China; Orthopaedic Medical Research Center , Dalian University, Dalian, Liaoning 116001, China; Department of Breast Oncology, Affiliated Zhongshan Hospital of Dalian University, Dalian, Liaoning 116001, China; Orthopaedic Medical Research Center , Dalian University, Dalian, Liaoning 116001, China; Department of Orthopedics, Affiliated Zhongshan Hospital of Dalian University, Dalian, Liaoning 116001, China; Department of Orthopedics, Affiliated Zhongshan Hospital of Dalian University, Dalian, Liaoning 116001, China; Orthopaedic Medical Research Center , Dalian University, Dalian, Liaoning 116001, China; Zhongshan Clinical College, Dalian University, Dalian, Liaoning 116001, China; Department of Orthopedics, Affiliated Zhongshan Hospital of Dalian University, Dalian, Liaoning 116001, China

**Keywords:** tumor microenvironment, immunotherapy, tissue engineering repair, postoperative defect in osteosarcoma, bone regeneration, antitumor

## Abstract

Advances in orthopedic biomaterials have significantly improved the bone regeneration domain; however, postoperative bone defect repair after bone tumor resection—particularly for osteosarcoma, a common primary malignant bone tumor in children and adolescents—remains a critical exception. Osteosarcoma’s aggressiveness, early metastatic propensity and immunosuppressive tumor microenvironment (OS-TME) limit conventional therapies, yielding poor prognosis in recurrence or metastasis cases. Although traditional tissue engineering promotes bone regeneration, it inadvertently sustains tumor growth, recurrence and metastasis via cytokines such as TGF-β1, VEGF and BMP-2. Mesenchymal stem cells and induced pluripotent stem cells worsen this by inducing M2 macrophage polarization, creating a tumor-supportive immune niche. Our prior research on biodegradable magnesium for bone repair showed Mg^2+^ enhances osteogenesis by activating PI3K/AKT via the TRPM7 channel; yet, TRPM7 as an oncogene links to tumor invasion and metastasis, posing risks for magnesium-based scaffolds in osteosarcoma defect repair. Thus, traditional tissue engineering’s three core elements (seed cells, scaffolds, cytokines) fail to meet dual needs of anti-tumor efficacy and bone regeneration in post-tumor defects. Recent tumor immunology breakthroughs have driven immuno-tissue engineering’s rise, offering new opportunities for osteosarcoma defect repair. Still, limited understanding of OS-TME mechanisms hinders clinical translation. This review delineates OS-TME’s immune landscape, covering immune cells (e.g. TAMs, Tregs, myeloid-derived suppressor cells), checkpoints (e.g. PD-1, CTLA-4, CD47) and immunotherapies and explores their tissue engineering integration through sequential release, spatiotemporal targeting, immune modulation and multimodal analyses (e.g. single-cell RNA sequencing, spatial transcriptomics) to optimize material design. We propose immune modulation as a novel ‘fourth element’ in tissue engineering, beyond the conventional seed cells, scaffolds and cytokines framework. This exposition lays a theoretical foundation for osteosarcoma immuno-tissue engineering repair and inspires innovative biomaterial solutions.

## Graphical abstract

**Graphical Abstract:**
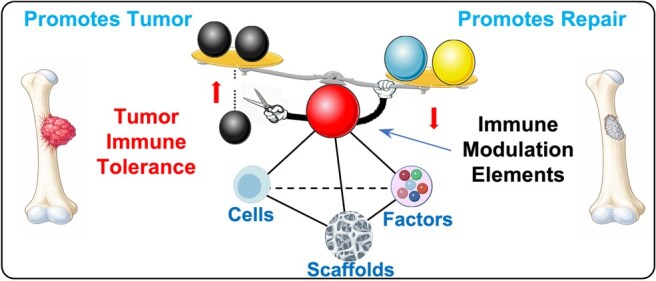

## Introduction

Osteosarcoma is the most common primary malignant bone tumor and predominantly affects children and adolescents. It can arise in any bone but most frequently involves the distal femur and proximal tibia [1]. The current standard of care for osteosarcoma primarily comprises surgical resection, neoadjuvant chemotherapy and adjuvant chemotherapy [2]. Since the widespread clinical adoption of neoadjuvant and adjuvant chemotherapy in the 1980s, the five-year survival rate for patients with localized osteosarcoma has risen to approximately 60% [3]. Nevertheless, owing to the highly aggressive nature of osteosarcoma and its propensity for early pulmonary metastasis, the 5-year survival rate has not improved substantially even nearly 40 years after adjuvant chemotherapy entered routine clinical use [4]. Even with conventional treatment, the mortality rate among patients with metastatic or recurrent osteosarcoma remains as high as 75% [5].

To ensure complete tumor clearance in clinical practice, osteosarcoma surgery typically requires wide resection of the lesion together with surrounding bone tissue [6]. The resulting segmental bone defects can severely compromise limb function and may lead to permanent disability. Traditional reconstructive approaches can partially restore skeletal integrity and function; however, in the context of postoperative chemotherapy, they are still limited by delayed bone healing and the inability to eradicate residual tumor cells [7]. In recent years, tissue engineering has offered new avenues for bone repair and regeneration. Through the synergistic use of scaffold materials and bioactive factors, tissue engineering can markedly accelerate osteogenesis [8]. However, these same strategies may carry unintended risks: multiple released cytokines [such as vascular endothelial growth factor (VEGF), bone morphogenetic protein-2 (BMP-2) and transforming growth factor-β1 (TGF-β1)] may indirectly stimulate the growth and dissemination of residual tumor cells [9–11]. Therefore, in the context of postresection bone defect repair for osteosarcoma, tissue engineering alone is unlikely to simultaneously promote bone regeneration and suppress tumor relapse, highlighting the urgent need to integrate antitumor modalities to enhance oncologic control.

Immunotherapy has recently achieved major breakthroughs in several malignancies, opening new possibilities for cancer treatment. For example, chimeric antigen receptor T-cell (CAR-T) therapy has substantially improved overall survival with a favorable safety profile in patients with B-cell acute lymphoblastic leukemia [12]. In addition, immune checkpoint inhibitors (such as the anti-CTLA-4 antibody ipilimumab and the anti-PD-1 antibody pembrolizumab) have been approved by the U.S. Food and Drug Administration (FDA) for advanced melanoma [13], and their remarkable clinical efficacy underscores the potential of immunotherapy in solid tumors.

However, compared with that in hematologic malignancies, immunotherapy in osteosarcoma still faces formidable challenges, largely because of the unique immunosuppressive tumor microenvironment, which substantially decreases the efficacy of approaches such as checkpoint blockade [14]. Consequently, overcoming immune evasion and remodeling the osteosarcoma tumor microenvironment (TME) have become key to improving therapeutic responses. Among the diverse immunoregulatory components, tumor-associated macrophages (TAMs)—a major cellular constituent of the immunosuppressive TME—play central roles in osteosarcoma initiation, progression and response to immunotherapy [15]. TAMs exhibit marked plasticity, with phenotypes and functions shaped by multiple signals. Leveraging this plasticity to target and reprogram TAMs from immunosuppressive to immunostimulatory states holds promise for relieving local immune suppression, enhancing immune recognition and cytotoxicity against tumor cells, and thereby improving the overall efficacy of immunotherapy [16].

In light of the limited progress with conventional treatment paradigms and the inherent risks of traditional reconstruction and tissue engineering-only strategies for postresection defects, we propose the integration of tissue engineering with immunotherapy to simultaneously promote bone repair and suppress residual tumor growth, offering a new reconstructive strategy after osteosarcoma surgery. This review aims to (i) delineate the characteristics of the osteosarcoma microenvironment (including immune cell constituents and immune checkpoints) and their mechanistic roles in tumor initiation and progression and (ii) summarize the current status and clinical advances of immunotherapy in osteosarcoma, thereby providing a theoretical foundation for combining tissue engineering with immunotherapy in the management of this disease.

## Characteristics of the osteosarcoma immune microenvironment

The tumor microenvironment of osteosarcoma (OS-TME) is a complex ecosystem composed primarily of osteosarcoma cells together with surrounding nonmalignant components [17]. It includes malignant cells, immune cells, stromal cells and the extracellular matrix; among these, immune cells play pivotal roles in osteosarcoma progression, immune evasion and therapeutic responsiveness [18, 19]. The immune compartment is dominated by macrophages and T lymphocytes, in addition to dendritic cells, natural killer (NK) cells and B lymphocytes also present [17]. Collectively, these immune cell populations constitute a dynamic regulatory network that participates in immune surveillance and tumor clearance but can be tumor-instructed and functionally reprogrammed to promote tumor initiation, progression, metastasis and immune escape [20].

Osteosarcoma is typically classified as an ‘immune-cold’ tumor characterized by a weak immune response and a highly immunosuppressive tumor microenvironment, particularly marked by the absence of CD8^+^ T cell and NK cell infiltration [21, 22]. This environment is often accompanied by high infiltration of immunosuppressive cells, such as myeloid-derived suppressor cells (MDSCs), regulatory T cells (Tregs) and M2 macrophage-type tumor-associated macrophages (TAMs), which further exacerbate the immunosuppressive status of the tumor microenvironment and promote immune evasion [23].

Moreover, immune checkpoint molecules such as PD-1, CTLA-4 and CD47 play pivotal roles in immune evasion mechanisms in osteosarcoma. These molecules suppress T-cell activity by binding to their ligands, thereby weakening T-cell-mediated immune surveillance of tumor cells [24]. These immune checkpoints not only limit the function of CD8^+^ T cells but also regulate the activity of immunosuppressive cells, further enhancing tumor immune escape [25, 26].

Thus, immune cells and immune checkpoint molecules within the tumor microenvironment are key regulatory factors in the initiation, progression and immune evasion of osteosarcoma. These immune cells, in particular, serve as critical determinants of the osteosarcoma immune microenvironment, shaping its immunosuppressive nature and influencing tumor dynamics through their interactions and functional states. A deeper understanding of these mechanisms, especially the specific characteristics and behaviors of immune cells within the osteosarcoma environment, is essential not only as the key to advancing immunotherapy for this tumor type but also as a breakthrough point for immuno-tissue engineering approaches, enabling integrated strategies that combine targeted immunomodulation with tissue regeneration for postsurgical bone defect repair and enhanced therapeutic outcomes.

## Characteristics of immune cells in the tumor immune microenvironment

**Figure 1:**
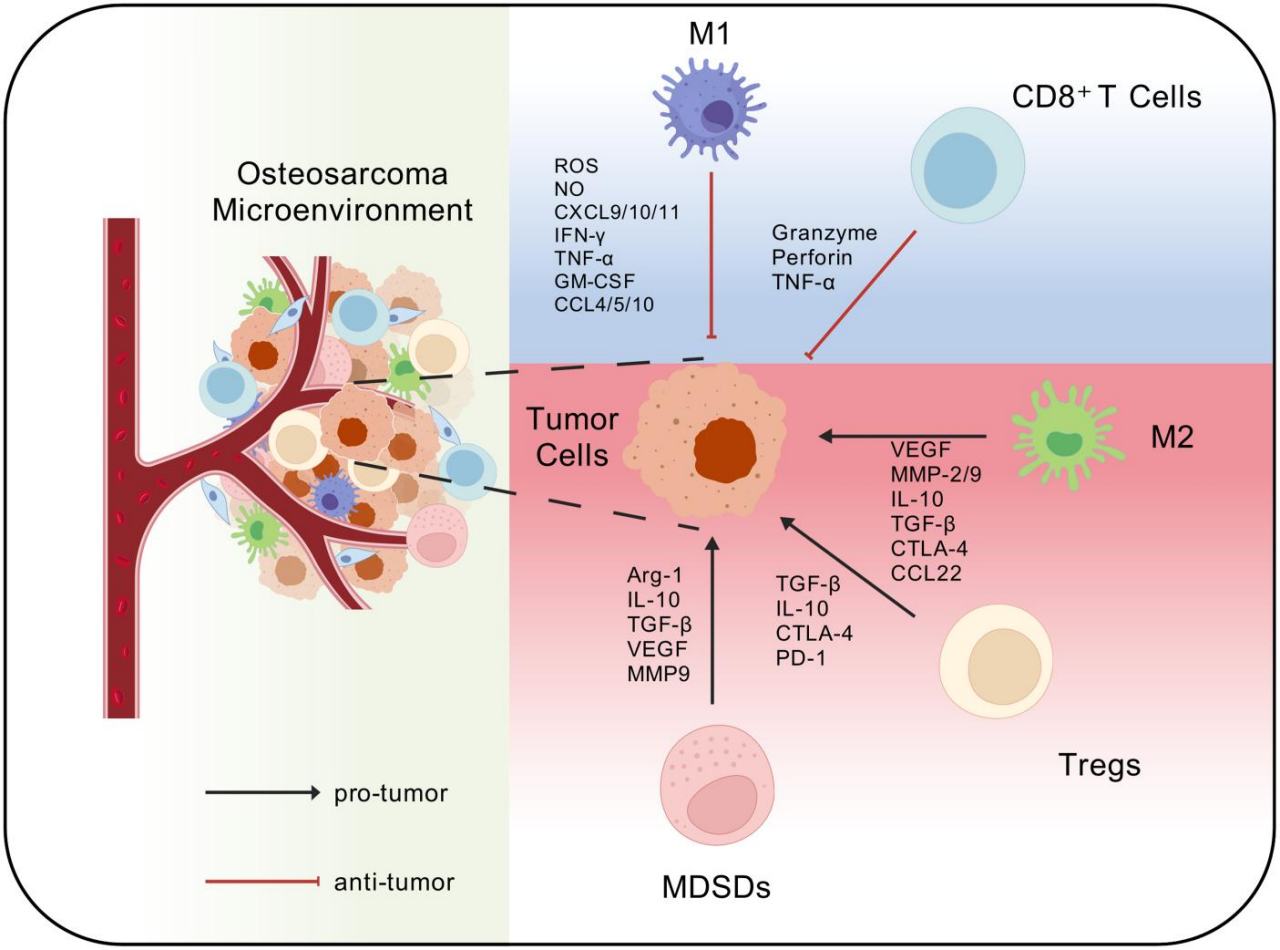
Immune-cell composition of the osteosarcoma tumor microenvironment (OS-TME). The OS-TME comprises multiple immune populations that collectively shape tumor initiation, progression, immune evasion and metastasis. Functionally, these cells can be grouped into antitumor and protumor subsets. (**A**) Antitumor immune cells include CD8^+^ T cells, which kill tumor cells through antigen recognition or TNF-α-mediated apoptosis, and M1 macrophages, which restrain tumor growth via phagocytosis, cytotoxic effector molecules and enhanced T-cell-mediated immunity. (**B**) Protumor immune cells include Tregs that suppress CD8^+^ T-cell function, M2 macrophages that promote proliferation, angiogenesis, invasion/metastasis and dampen antitumor immunity and MDSCs that support tumor growth/angiogenesis while inhibiting T-cell and NK-cell activity and reinforcing Tregs/TAMs functions. Created with BioGDP.com [27].

The growth of osteosarcoma is regulated by multiple immune cell populations within the osteosarcoma tumor microenvironment (OS-TME). Functionally, these cells can be broadly categorized into two groups: (i) antitumor immune cells [primarily CD8^+^ T cells and classically activated (M1) macrophages], which mediate immune surveillance and the clearance of malignant cells; and (ii) protumor immune cells [regulatory T cells (Tregs), alternatively activated (M2) macrophages and MDSCs], which play pivotal roles in tumor initiation, progression, metastasis and immune escape (Figure 1). Immune cells exhibit both antitumor and protumor roles in the tumor microenvironment. Therefore, a detailed understanding of the mechanisms and cross-talk among these immune subsets in the OS-TME provides a critical theoretical basis for the development of new therapeutic strategies that integrate immune cell modulation with tissue engineering approaches (Table 1).

**Table 1:** Functions of immune cell in the tumor microenvironment.

Antitumor/Protumor	Cell type	Functions in the tumor microenvironment	Ref.
Antitumor	CD8^+^ T cells	Kill tumor cells by releasing perforin and granzymes or by inducing apoptosis via TNF-α secretion.	[28, 29]
Protumor	Tregs	Treg cells inhibit CD8⁺ T cells and APCs by secreting TGF-β and IL-10, and further suppress CD8⁺ T cells function through high expression of CTLA-4 and PD-1.	[30, 31]
Antitumor	DCs	Dendritic cells are the most potent antigen-presenting cells, capable of presenting tumor-specific antigens to cytotoxic T cells, thereby activating cellular immunity to kill tumor cells.	[32]
Antitumor	M1 macrophages	M1 macrophages suppress tumor cells through phagocytosis, cytotoxic molecule-mediated killing and T-cell-mediated immune cytotoxicity.	[33–36]
Protumor	M2 macrophages	M2 macrophages promote tumor progression via tumor proliferation, angiogenesis, invasion and metastasis, and immune evasion.	[37–41]
Protumor	MDSCs	MDSCs promote tumor growth, angiogenesis, inhibit T-cell and NK cell activity, and enhance the function of Tregs and TAMs.	[42–44]

### T cells

T cells play a central role in antitumor immunity, particularly during immune surveillance and the elimination of malignant cells [45]. Within the tumor microenvironment, the most representative subsets are CD8^+^ T cells and regulatory T cells (Tregs).

CD8^+^ T cells are the principal effector lymphocytes of the adaptive immune response and are capable of specifically killing infected or malignant cells. Upon recognition by the T-cell receptor (TCR) of tumor-associated antigenic peptides presented on major histocompatibility complex class I (MHC I) molecules, CD8^+^ T cells become activated and mediate cytotoxicity either by releasing perforin and granzymes to directly lyse cancer cells or by secreting tumor necrosis factor-α (TNF-α) to induce apoptosis [28, 46–48]. However, single-cell RNA sequencing (scRNA-seq) analyses of osteosarcoma tumor microenvironments have revealed varying degrees of CD8^+^ T cells exhaustion, which directly impairs their effector function [49].

In contrast, Tregs maintain immune tolerance and prevent excessive immune activation under physiological conditions. In the tumor microenvironment, Tregs suppress the activity of CD8^+^ T cells and antigen-presenting cells (APCs) through the paracrine secretion of immunosuppressive cytokines such as transforming growth factor-β (TGF-β) and interleukin-10 (IL-10) [28, 50]. Tregs also express high levels of immune checkpoint molecules, including CTLA-4 and PD-1, which further attenuate CD8^+^ T cells function [31]. In 2023, Cheng *et al.* [51] reported that abundant Tregs infiltrating osteosarcoma tissue promote tumor progression by activating key pathways (oxidative phosphorylation, angiogenesis and mTORC1) and by engaging in cross-talk with osteoblasts, endothelial cells and myeloid cells through the CXCL–CXCR4 and CXCL12–TGFB1 signaling axes.

Despite the central effector role of T cells, the ‘immune-cold’ phenotype of osteosarcoma cells diminishes CD8^+^ T cells infiltration/function and increases the number of regulatory T cells (Tregs), promoting tumor growth and immune evasion. Thus, enhancing CD8^+^ T cells cytotoxicity and curbing Treg infiltration are major immunotherapeutic priorities.

#### Therapeutic approaches

The primary aim of T-cell-based immunotherapies for osteosarcoma, which include chimeric antigen receptor T-cell (CAR T) strategies, is to enhance CD8^+^ T cells activity or limit Treg infiltration.

With respect to CD8^+^ T cells-directed approaches, Hennessy *et al.* [52] reported that bempegaldesleukin (BEMPEG; NKTR-214), a first-in-class CD122-preferential IL-2 pathway agonist, produced robust antitumor effects in murine osteosarcoma models. Treatment markedly increased the intratumoral accumulation of effector T cells and NK cells without promoting Treg expansion, which suppressed primary tumor growth and reduced pulmonary and skeletal metastatic relapse. In 2024, He *et al.* [53] subsequently developed an injectable hydrogel microsphere-integrated training platform (MS-ITC) that incorporates cytokines such as IL-7 and IL-15. This platform significantly increased CD8^+^ T cells expansion and thereby enhanced antitumor efficacy against osteosarcoma. The innovative combination of biomaterials with cytokine delivery offers a new strategy for osteosarcoma immunotherapy.

With respect to Treg-targeted immunotherapy, Yoshida *et al.* [54] first demonstrated that 4H2 (a PD-1 antibody) can inhibit Treg infiltration in osteosarcoma, yielding antitumor activity. In addition, Fujiwara *et al.* [55] reported the therapeutic potential of PLX3397 (a CSF1 receptor inhibitor) in osteosarcoma, which not only increased CD8^+^ T cells infiltration in both primary and metastatic tumor microenvironments but also depleted Tregs. These studies provide compelling evidence to support Treg-directed strategies.

CAR T-cell therapy has likewise shown notable activity in osteosarcoma. Majzner *et al.* [56] reported that B7-H3 CAR-T cells mediate potent *in vivo* antitumor effects, leading to the regression of established solid tumors, including osteosarcoma, in xenograft models. Building on these findings, Lake *et al.* [57] reported that sublethal irradiation induces IL-8 production by sarcoma cells. They engineered B7-H3 CAR-T cells to express the IL-8 receptor CXCR2 and demonstrated improved binding to IL-8-high tumors, enhanced T-cell metabolism and pronounced tumor regression.

In osteosarcoma, T-cell-based immunotherapies have shown clear antitumor activity in early studies (Table 2). Coordinating these approaches with tissue-engineered bone repair for postoperative defect reconstruction (e.g. combining local biomaterial delivery with immune modulation) may simultaneously promote bone regeneration and functional recovery while suppressing recurrence, thereby offering a new direction for osteosarcoma treatment.

### Myeloid-derived suppressor cells

MDSCs are a heterogeneous population that play a pivotal role in the immune system. Although their precise definition remains debated [58], they are unequivocally characterized by potent immunosuppressive and protumorigenic activities [59]. Under physiological conditions, immature myeloid cells differentiate into granulocytes, macrophages and dendritic cells, thereby participating in host defense against pathogens. In disease states such as tumors, this normal differentiation program is impeded [60]. Persistent inflammation-related growth factors and cytokines within the tumor milieu drive the activation and accumulation of MDSCs in the bone marrow and tumor microenvironment [59, 61].

MDSCs promote tumor growth and suppress antitumor immunity through multiple mechanisms, including immunosuppressive and nonimmune functions. Immunosuppression is a hallmark of MDSC biology. Within the tumor microenvironment, MDSCs directly inhibit T-cell and NK-cell activity by expressing arginase-1 (Arg-1) and inducing the production of nitric oxide (NO) and reactive oxygen species (ROS), thereby undermining cytotoxic antitumor responses [42, 62]. In addition, MDSCs secrete high levels of IL-10 and TGF-β, which induce regulatory T cells (Tregs) and M2-polarized tumor-associated macrophages (TAMs), further suppressing antitumor immunity [43, 63].

Beyond immunosuppression, MDSCs contribute to tumor initiation and progression by secreting proangiogenic factors such as VEGF and MMP-9, thereby facilitating neovascularization [64]. In addition, they promote and maintain the cancer stem cell pool via the IL-6/STAT3 and NO/NOTCH pathways, which further supports tumor growth and metastatic dissemination [44, 65].

#### Therapeutic approaches

MDSCs play pivotal roles in tumor immune evasion and tumor progression, and they engage in close crosstalk with other immunosuppressive populations (including TAMs and Tregs). Accordingly, targeting MDSCs to blunt their protumor functions and restore antitumor immunity is a key therapeutic strategy for osteosarcoma. Using a murine model, Hu *et al.* [66] demonstrated that apatinib and/or antigen-specific dendritic cell-T-cell (DC-T) therapy could reduce the proportion of MDSCs, ultimately enhancing immunologic activity within the tumor microenvironment (TME). Subsequently, Shrestha *et al.* [67] reported that the STAT3 inhibitor WP1066 decreases MDSC survival and proliferation as well as their suppressive effects on CD8^+^ T cells. Collectively, these findings highlight the substantial therapeutic potential of MDSC-directed interventions in osteosarcoma; integrating such approaches with tissue engineering may offer new opportunities for postoperative bone defect repair and adjuvant immunotherapy (Table 2).

**Table 2:** Immune cell-targeted therapy for osteosarcoma.

Materials and/or factors	Target	Cells	Animals	Research results	Time	Country	Ref.
Bempegaldesleukin	CD8⁺ T cells, NK cells	K7M2, K7M3	female BALB/c mice	Bempegaldesleukin enhances intratumoral effector T and NK cell infiltration while sparing Tregs, thereby suppressing primary tumor growth and metastatic relapse.	2021	USA	[52]
Anti-CD3/CD28 antibodies and IL-7/IL-15 albumin nanoparticles conjugated to hydrogel microspheres	CD8⁺ T cells	K7M2	BALB/c mice	MS-ITC enhances antitumor efficacy by significantly increasing CD8^+^ T cells and memory T cells, resulting in more than 95% inhibition of primary osteosarcoma growth.	2024	China	[53]
Anti-PD-1 antibody (4H2)	Tregs	LM8, HOS, SaOS-2, 143B	Male C3H/HeSlc mice	Anti-PD-1 antibody suppresses the infiltration of Tregs into tumors and exerts an antitumor effect.	2020	Japan	[54]
B7-H3 CAR T cells	CAR T cells	MG63.3, K562, EW8, NALM6-GL, DAOY, D283, D425293GP, 293 T	NOD.Cg-Prkdcscid Il2rgtm1Wjl/SzJ)	B7-H3 CAR T cells mediate significant antitumor activity in vivo, causing regression of established solid tumors in xenograft models, including osteosarcoma, medulloblastoma, and Ewing sarcoma.	2019	USA	[56]
B7-H3 CAR T cells that also express the interleukin-8 (IL-8) receptor, CXCR2	CAR T cells	HOS, U2OS, RH30, JR1, RD, 293GP, HEK293T	NOD-scid IL2Rgnull (NSG) mice	IL-8 is expressed by RMS and OS, and expression significantly increases after radiation. Overexpression of an IL-8 receptor, CXCR2, on B7-H3 CAR T cells enhances homing into IL-8 expressing tumors, augments T-cell metabolism and leads to significant tumor regression.	2024	USA	[57]
Apatinib and antigen-specific dendritic cell (DC)-T	MDSCs	HOS-8603	Male G57BL/6 mice	The ratio of MDSCs is reduced by apatinib or antigen-specific DC-T-cell treatment, while it remained at a lower level with the combination treatment.	2024	China	[66]
WP1066（STAT3 inhibitor）	MDSCs	MG63, K7M3	BALB/c female mice	WP1066 reduces the survival and proliferative capacity of myeloid-derived suppressor cells (MDSCs) and diminishes their suppressive activity toward CD8^+^ T cells.	2025	USA	[67]

### Macrophages

Macrophages, which are essential immune effectors in humans, constitute the most abundant infiltrating immune population within the osteosarcoma tumor microenvironment (TME) [68]. These macrophages, once recruited into the TME, are referred to as tumor-associated macrophages (TAMs) and arise predominantly from circulating monocytes. Immunoregulatory factors produced within the TME—such as CSF-1 and CCL2—actively recruit monocytes to tumor sites, where they differentiate into TAMs and participate in immune modulation [69, 70]. In addition, a subset of macrophages derives from embryonically seeded tissue-resident lineages [71]. Beyond their roles in physiologic tumor immunosurveillance, TAMs critically influence osteosarcoma progression and immune evasion.

Functionally, macrophages are broadly classified into two subsets, M1 and M2. M1 (‘classically activated’) macrophages exhibit robust proinflammatory and antitumor activities, whereas M2 (‘alternatively activated’) macrophages are involved primarily in immunosuppression and tissue repair and can promote tumor progression and immune escape [33]. Macrophages display marked plasticity, with polarization states shaped by microenvironmental cues [72]. Cytokines and chemokines such as IFN-γ, IL-1, IL-12, CXCL9, CXCL10 and CXCL11 favor M1 polarization, whereas IL-4, IL-10, CCL2/3/4/5, CXCL12 and VEGF drive polarization toward the M2 phenotype [35, 73, 74]. Given this duality, macrophages may become pivotal targets for both immunotherapeutic strategies and tissue engineering-based repair in osteosarcoma.

#### M1 macrophages

M1 macrophages eliminate and restrain tumor cells through phagocytosis and cytotoxic effector mechanisms and by increasing T-cell-mediated cytotoxicity. Macrophages are the principal phagocytes that operate within tumors and are targeted by nonspecific, direct engulfment and antibody-dependent cellular phagocytosis (ADCP) [34].

**Figure 2:**
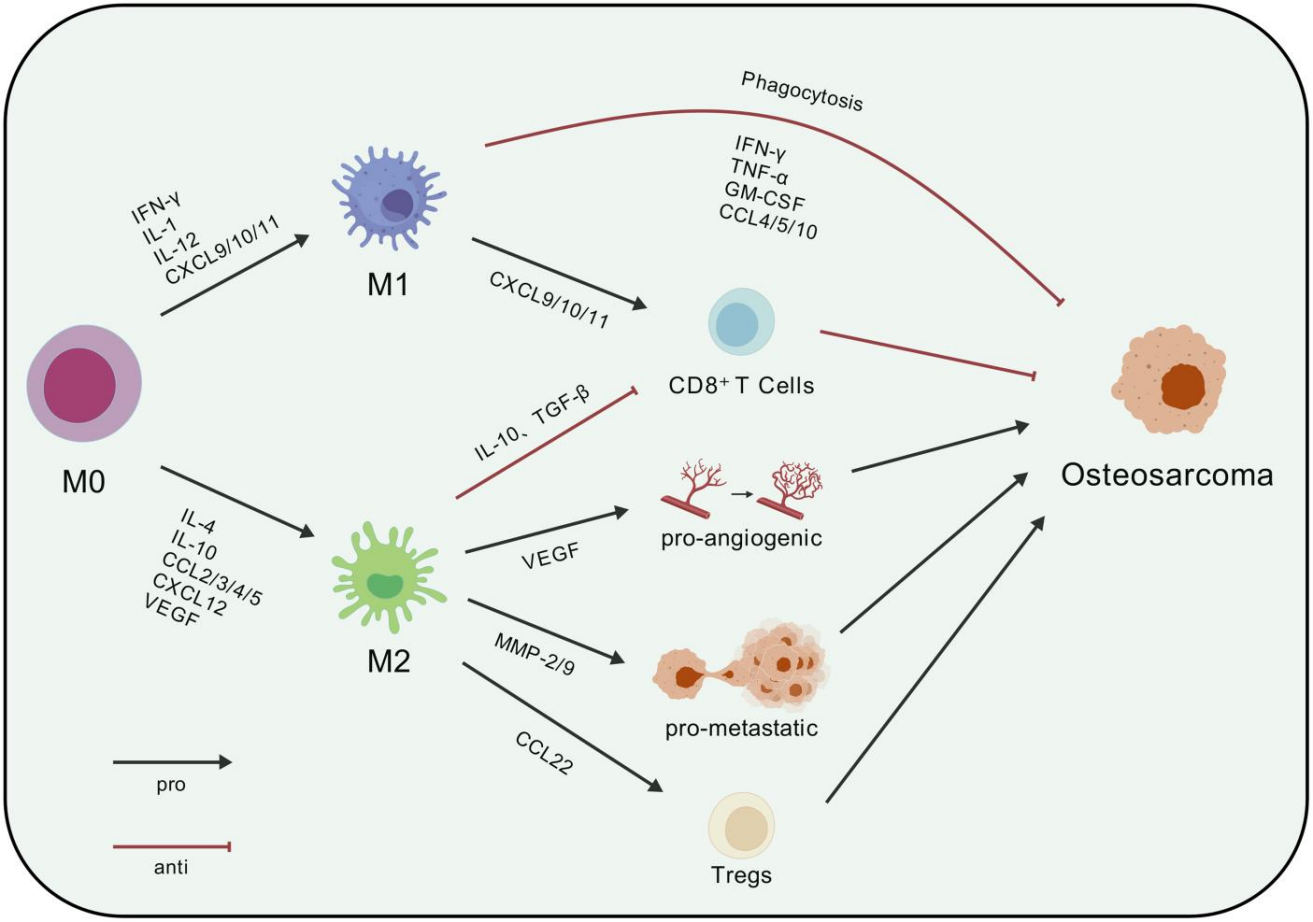
Polarization and functions of macrophage subsets in the osteosarcoma tumor microenvironment (OS-TME). Monocyte-derived M0 macrophages polarize toward M1 macrophages in response to IFN-γ/LPS or toward M2 macrophages upon IL-4/IL-10 signaling. M1 macrophages limit osteosarcoma progression through phagocytosis (including ADCP) and iNOS-dependent cytotoxicity (ROS/NO), and they recruit/activate CD8^+^ T cells via CXCL9/10/11 while releasing IFN-γ, TNF-α, GM-CSF and CCL4/5/10. In contrast, M2 macrophages suppress antitumor immunity and support tumor progression by secreting IL-10 and TGF-β, promoting angiogenesis via VEGF, driving invasion/metastasis via MMP-2/9 and recruiting Tregs through CCL22. Created with BioGDP.com [27].

Cytotoxic, molecular-mediated killing constitutes another major antitumor pathway of macrophages and includes both direct cytotoxicity and antibody-dependent cellular cytotoxicity (ADCC; Figure 2) [33]. Upon activation, M1 macrophages upregulate inducible nitric oxide synthase (iNOS) and NADPH oxidase, thereby generating reactive oxygen species (ROS) and nitric oxide (NO); these mediators directly damage tumor cell DNA and proteins, resulting in cytotoxic effects [36]. In parallel, M1 macrophages secrete a repertoire of chemokines—notably CXCL9, CXCL10 and CXCL11—that further recruit and activate NK cells and T cells. These activated lymphocytes can directly lyse tumor cells or release IFN-γ, TNF-α, GM-CSF and chemokines such as CCL4, CCL5 and CCL10 to further suppress tumor growth [35].

A phagocytosis checkpoint constrains this activity: the inhibitory receptor SIRPα on macrophages engages CD47 on target cells to suppress engulfment. In 2009, Majeti *et al.* [75] first demonstrated that acute myeloid leukemia cells evade macrophage phagocytosis by overexpressing CD47 to ligate SIRPα. The downregulation of L-type amino acid transporter 2 (LAT2) expression subsequently decreases CD47 expression on osteosarcoma cells, thereby increasing macrophage infiltration and enhancing phagocytosis [76]. Thus, therapeutically blocking CD47 to restore M1 macrophage-mediated phagocytosis has become an important strategy.

Nevertheless, osteosarcoma cells and their tumor microenvironment recruit macrophages and skew them toward the protumorigenic M2 phenotype, resulting in a relatively low proportion of antitumor M1 macrophages in osteosarcoma and limiting their efficacy [37]. Consequently, leveraging macrophage plasticity to reprogram the M1/M2 balance within the TME—and inhibiting the CD47–SIRPα axis to restore M1-associated antitumor functions—has become a key therapeutic objective in osteosarcoma.

#### M2 macrophages

M2-polarized macrophages play essential roles in anti-inflammatory responses, tissue repair and immune regulation [77]. During inflammation, M2 macrophages secrete factors such as IL-10 and TGF-β to suppress excessive inflammation—thereby limiting tissue damage—and on this basis promote tissue repair [78]. Current evidence indicates that macrophages can facilitate bone regeneration by regulating the recruitment and differentiation of mesenchymal stem cells (MSCs) [79]. However, M2 macrophages also exhibit protumorigenic activities and contribute to immune escape [37]. In the tumor microenvironment (TME), they promote tumor cell proliferation, angiogenesis, invasion and metastasis while suppressing antitumor immune responses (Figure 2).

Bidirectional interactions occur between tumor cells and macrophages. Studies have shown that tumor cells secrete CSF-1, which attracts intravascular monocytes to extravasate and accumulate around tumor foci; CSF-1 also directly stimulates macrophages to release EGF, which in turn augments tumor cell proliferation [38, 80]. In osteosarcoma, reports likewise demonstrate that osteosarcoma-derived migrasomes and exosomes can drive macrophage polarization toward the M2 phenotype, thereby enhancing osteosarcoma cell migration and invasion [37, 81]. Collectively, these findings indicate that tumor cells induce M2 polarization, and M2 macrophages reciprocally promote tumor cell proliferation and dissemination, establishing a malignant positive-feedback loop.

Tumor growth is highly dependent on angiogenesis, in which neovessels supply oxygen and nutrients to sustain further expansion, with hypoxia in the tumor microenvironment serving as a major driving force. Studies have shown that hypoxic conditions not only skew macrophage polarization toward a protumorigenic M2 state [39] but also stimulate M2 macrophages to produce VEGF, further promoting neovascularization within the TME [40]. Tumor invasion and metastasis also rely heavily on matrix metalloproteinases (MMPs) present in the TME. MMPs are hydrolytic enzymes capable of degrading the extracellular matrix (ECM) and basement membrane, thereby facilitating tumor cell invasion and dissemination [82]. It has been demonstrated that the paracrine secretion of MMP-2 and MMP-9 by M2 macrophages can disrupt the fibrous capsule of hepatocellular carcinoma, subsequently promoting tumor invasion and metastasis [83].

In addition to directly secreting immunosuppressive cytokines such as IL-10 and TGF-β to decrease CD8^+^ T cells activity, M2 macrophages can facilitate immune escape through other mechanisms. PD-L1 is highly expressed on M2 macrophages [84]; the binding of PD-L1 to the PD-1 receptor on T cells suppresses CD8^+^ T cells activation and induces apoptosis, thereby aiding tumor immune evasion [41, 85]. Moreover, M2 macrophages can express CTLA-4 and inhibit T cell activation by competitively binding B7 molecules on antigen-presenting cells, further contributing to immune escape [41]. In addition, M2 macrophages secrete the chemokine CCL22 to recruit regulatory T cells (Tregs) into the tumor milieu and Tregs themselves promote tumor immune evasion [72].

In summary, M2 macrophages play pivotal roles in tumor growth, metastasis and immune escape and have become a major focus of tumor immunology research. Nevertheless, M2 macrophages exhibit a pronounced duality in the development and treatment of osteosarcoma. During disease progression, they promote osteosarcoma cell proliferation, dissemination and immune evasion, thereby accelerating tumor progression. In contrast, during the postoperative repair of bone defects, M2 macrophages support bone regeneration and expedite osseous healing. Therefore, the precise temporal and phenotypic control of M2 polarization—namely, suppressing protumor functions during tumor-clearing phases while enhancing proregenerative activities during bone-repair phases—is a key challenge in osteosarcoma management and renders M2 macrophages as highly promising therapeutic targets.

#### Therapeutic approaches

Macrophages play a pivotal role in osteosarcoma immunotherapy. Within the osteosarcoma microenvironment, the high degree of tumor-associated macrophage (TAM) infiltration and the predominance of the M2 subset not only promote tumor growth but also facilitate immune evasion by tumor cells, thereby exacerbating malignant progression. Consequently, modulating macrophage polarization—particularly by reducing the accumulation of M2 macrophages or inducing their repolarization toward the M1 phenotype—has become a promising therapeutic strategy.

First, most TAMs originate from bone marrow-derived monocytes [71]. Therefore, reducing macrophage recruitment can decrease the number of M2 macrophages by limiting their trafficking to and retention within the tumor microenvironment. Colony-stimulating factor 1 (CSF-1) was the first factor demonstrated to promote monocyte migration into the tumor milieu. In animal studies, Jeffrey W. Pollard and colleagues reported that CSF-1 recruits TAMs and promotes tumor progression and pulmonary metastasis [86]. In 2019, the CSF-1 receptor inhibitor PLX3397 received FDA approval for the treatment of tenosynovial giant cell tumors. Subsequent studies reported that PLX3397 reduces the infiltration of TAMs and Tregs while enhancing CD8^+^ T cells infiltration, thereby suppressing primary osteosarcoma growth and lung metastasis and prolonging metastasis-free survival [55]. In addition to CSF-1, CCL2 can recruit monocytes into the tumor microenvironment. Kondo *et al.* [87] demonstrated that osteosarcoma cells promote the accumulation of M2 macrophages in the lung via CCL2 secretion, thereby driving pulmonary metastasis of osteosarcoma cells. Moreover, Regan *et al.* [88, 89] reported that losartan significantly inhibits monocyte recruitment by noncompetitively blocking CCL2-induced ERK1/2 activation and that its combination with toceranib results in notable antitumor activity in canine metastatic osteosarcoma.

Second, direct regulation of macrophage polarization is critical. In response to microenvironmental cues, macrophages can be polarized into antitumor M1 or protumor M2 phenotypes. Inducing the conversion of M2 macrophages toward the M1 state—via small molecules, antibodies or gene-based approaches—is promising for restoring immune recognition and clearance of osteosarcoma. Zhou *et al.* [90] reported that all-trans retinoic acid (ATRA) exerts antimetastatic activity in osteosarcoma by inhibiting M2 polarization. In 2022, Huang *et al.* [91] reported that METTL14 in combination with the TLR4 agonist RS09 induces macrophage polarization toward the M1 phenotype and significantly suppresses tumor growth. Other studies have shown that curcumol inhibits M2 polarization in osteosarcoma tissue and, when combined with cisplatin (CDDP), markedly enhances the therapeutic efficacy of CDDP [92]. In 2023, Richert *et al.* [93] demonstrated that the TLR4 agonist liposomal monophosphoryl lipid A (Lipo-MP-LPS) reprograms protumor M2 macrophages into anticancer M1 macrophages, remodels the osteosarcoma tumor microenvironment, promotes T-cell recruitment and ultimately enhances tumor cell death. In 2024, Wu *et al.* [94] reported that CuB reduces M2 polarization by inhibiting the PI3K/AKT pathway, thereby suppressing osteosarcoma progression *in vivo*. Additionally, codelivery of chemotherapeutic doxorubicin (DOX) and the immunomodulator zoledronic acid (ZA) using aluminum-hydroxide nanosheets (nAl) induces macrophage polarization toward the M1 phenotype and augments antiosteosarcoma activity [95].

In summary, intervening in the recruitment and polarization of macrophages to overcome the formation of an immunosuppressive tumor microenvironment may provide a novel strategy for the treatment of osteosarcoma (Table 3). Whereas prior work—driven by antitumor imperatives—has largely focused on uniformly suppressing M2 polarization, we propose a temporally staged polarization strategy: suppression of the protumor functions of M2 macrophages during the tumor clearance phase and activation of their prorepair functions during the bone-healing phase. This strategy provides a new perspective and direction for osteosarcoma immunotherapy.

**Table 3:** Macrophage-targeted drugs for osteosarcoma.

Drug	Targeted target	Mechanism of action	Research results/Benefits	Time	Country	Ref.
Pexidartinib (PLX3397)	CSF-1/CSR-1pathway	Inhibits the CSF-1R pathway	In the osteosarcoma mouse model, PLX3397 inhibits primary tumor growth and lung metastasis, extending metastasis-free survival. PLX3397 also depletes TAMs, reduces Tregs, and enhances CD8^+^ T cells migration and infiltration into tumors.	2021	USA	[55]
Losartan	CCL2/CCR2pathway	Inhibition of CCL2-induced ERK signaling	Losartan significantly inhibits monocyte recruitment through noncompetitive inhibition of CCL2-induced ERK1/2 activation and, when combined with toceranib, demonstrates notable antitumor activity in dogs with metastatic osteosarcoma.	2022	USA	[88, 89]
ATRA	MMP12	Inhibition of M2 macrophage polarization and reducing M2 macrophage secretion of MMP12	All-trans retinoic acid (ATRA) inhibits M2 macrophage polarization in vitro and suppresses M2 macrophage-induced osteosarcoma migration. *In vivo*, it reduces osteosarcoma metastasis and inhibits M2 macrophage polarization in metastatic lesions.	2017	China	[90]
Curcumol	PI3K/AKT pathway	NA	Curcumol suppresses the polarization of M2-type macrophages within osteosarcoma tissue, thereby potentially modulating the tumor immune microenvironment.	2022	China	[92]
CuB	PI3K/AKT pathway	Inhibition of the PI3K/AKT pathway	In mouse models, CuB not only reduces the number of M2-polarized macrophages but also significantly decreases tumor weight and the number of pulmonary metastases.	2024	China	[94]

## Characteristics of immune checkpoints in osteosarcoma

In addition to the profoundly immunosuppressive microenvironment driven by the heavy infiltration of regulatory immune cells, immune checkpoints play a pivotal role in the immune evasion of osteosarcoma. In osteosarcoma, multiple checkpoints—such as programmed cell death protein 1 (PD-1) and cytotoxic T-lymphocyte-associated antigen 4 (CTLA-4)—are upregulated, and these pathways maintain immune tolerance by suppressing T-cell activity, thereby enabling tumor immune escape. In addition, the ‘don’ t-eat-me’ signal CD47 binds to signal regulatory protein-α (SIRPα) to inhibit macrophage phagocytosis, further promoting immune evasion.

### PD-1

PD-1 (CD279) is an inhibitory immune receptor that is expressed broadly on immune cells, including T cells and macrophages [96]. Its ligands, PD-L1 (B7-H1, CD274) and PD-L2 (B7-DC, CD273), are expressed on osteosarcoma cells and on antigen-presenting cells (APCs) within the tumor microenvironment (e.g. macrophages and dendritic cells) [97, 98].

**Figure 3:**
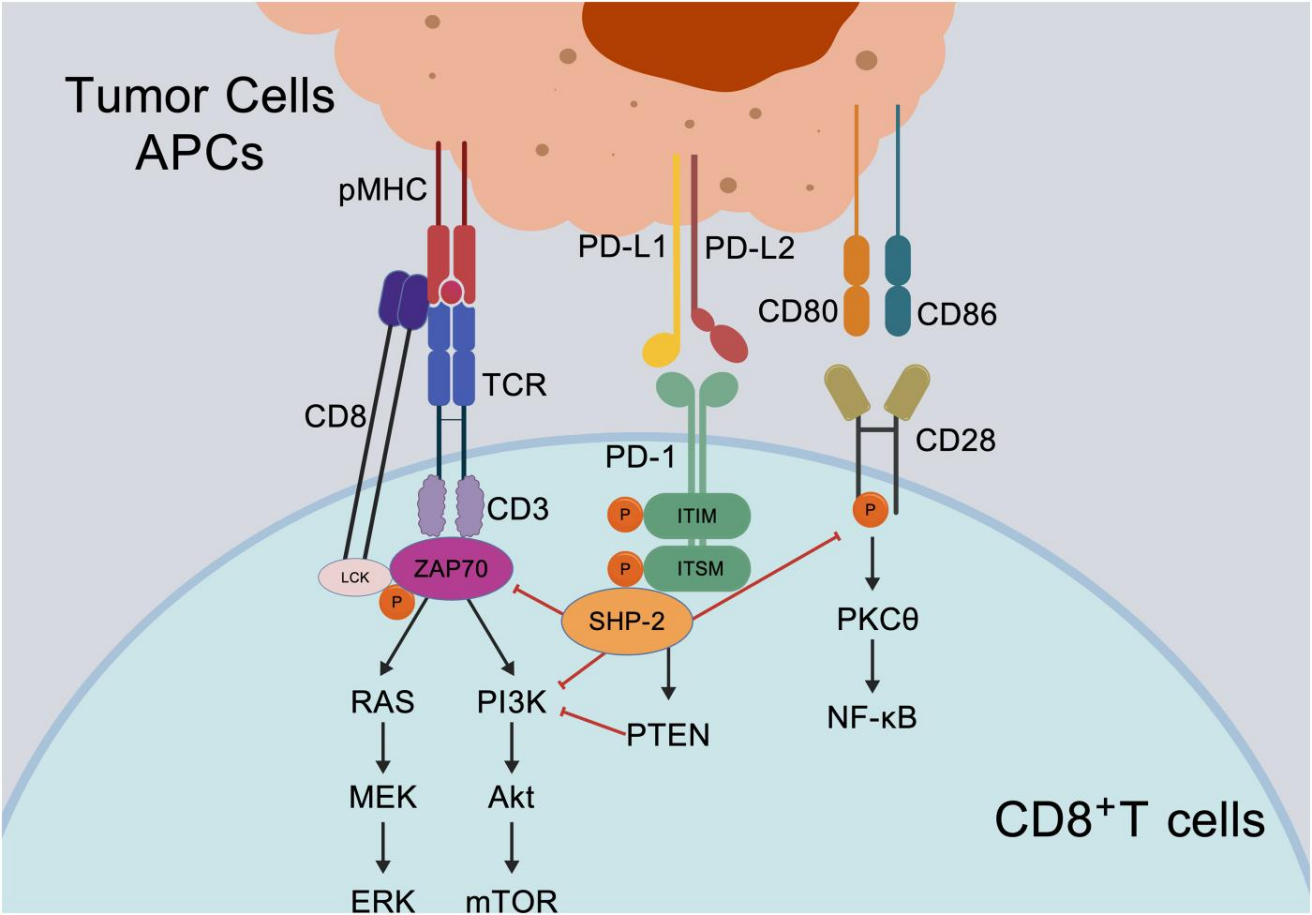
PD-1/PD-L1 Axis-mediated T cell inhibition in osteosarcoma. Binding of PD-1 on T cells to PD-L1/PD-L2 on osteosarcoma cells and antigen-presenting cells triggers phosphorylation of PD-1 cytoplasmic ITIM/ITSM motifs and recruitment of SHP-2. SHP-2 dephosphorylates CD28 and suppresses downstream PI3K–AKT and RAS–ERK signaling, leading to reduced T-cell proliferation, survival and effector function, thereby promoting T-cell exhaustion, immune evasion and metastasis. Created with BioGDP.com [27].

When PD-L1 on APCs engages PD-1 on activated T cells, tyrosine residues within the immunoreceptor tyrosine-based switch motif (ITSM) and immunoreceptor tyrosine-based inhibitory motif (ITIM) of PD-1 become phosphorylated, which leads to the recruitment and activation of SHP-2 [99–101]. SHP-2 attenuates downstream T-cell receptor (TCR) signaling by dampening key pathways—including the phosphoinositide 3-kinase (PI3K)–AKT axis and the extracellular signal-regulated kinase (ERK) cascade—thereby suppressing T-cell proliferation, activation, survival and cytotoxic function [102–104]. More recent work has further indicated that SHP-2 does not act primarily by directly targeting the TCR; rather, it mediates the dephosphorylation of the costimulatory receptor CD28, providing an additional mechanism for T-cell inhibition (Figure 3) [24].

Physiologically, the PD-1/PD-L1 pathway serves as a negative regulator of immune responses, preventing excessive activation and protecting host tissues from collateral damage [105, 106]. Osteosarcoma cells coopt this pathway to suppress antitumor immunity and facilitate immune escape. Zheng *et al.* [107] reported that PD-1 expression on T cells from patients with osteosarcoma is significantly upregulated, with even higher levels observed in those with metastatic disease.

### CTLA-4

Cytotoxic T-lymphocyte-associated antigen 4 (CTLA-4) is a key immune checkpoint molecule expressed on T cells, where it plays a central role in immune regulation [108]. By modulating T-cell activation, proliferation and effector function through multiple mechanisms, CTLA-4 prevents excessive immune responses and the development of autoimmunity.

**Figure 4:**
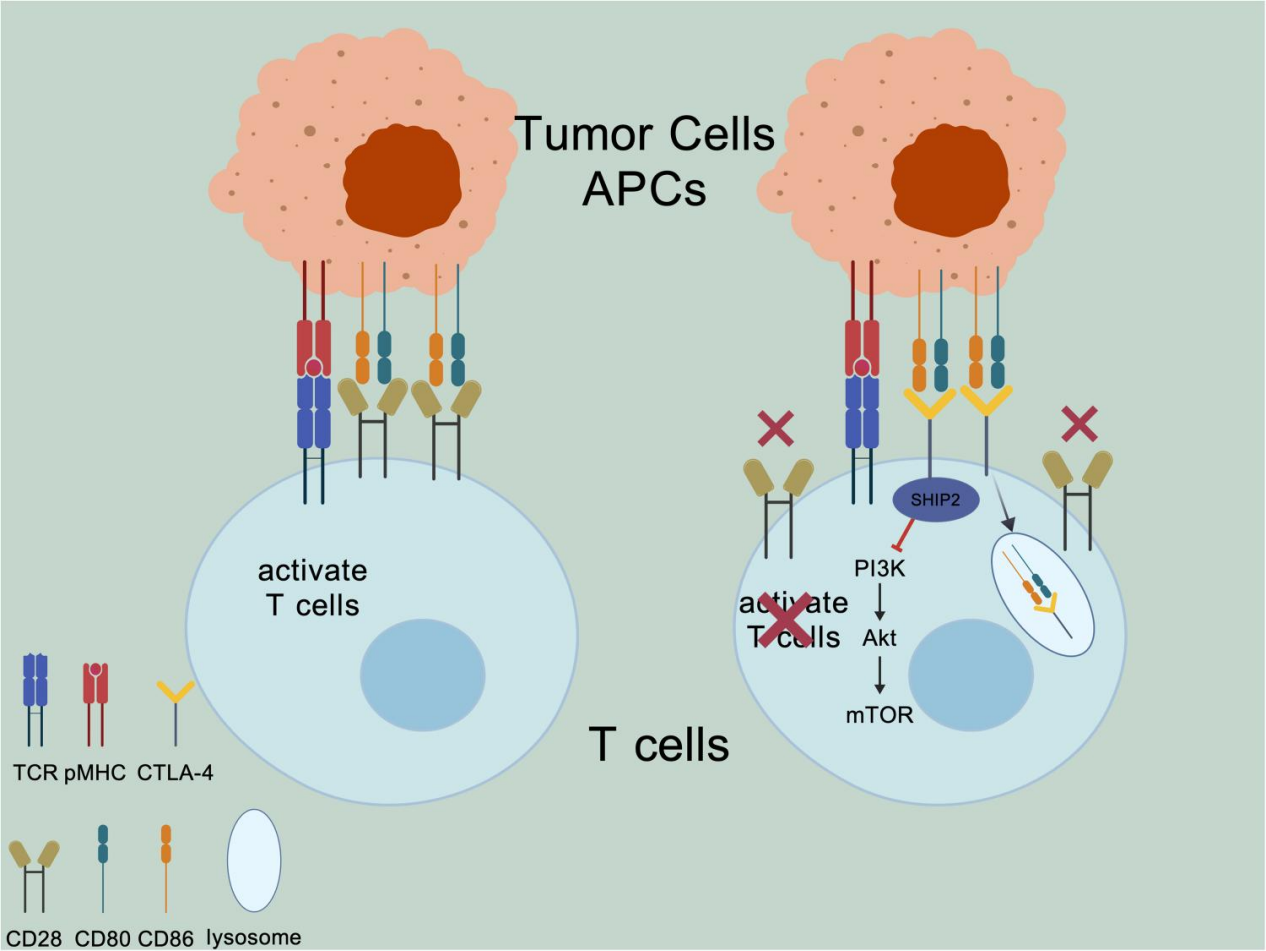
CTLA-4-Mediated regulation of early costimulatory checkpoints in osteosarcoma. CTLA-4 competes with CD28 for CD80/CD86 on antigen-presenting cells, weakening TCR-driven costimulatory signaling; it can also remove CD80/CD86 from the APC surface via trans-endocytosis. In addition, CTLA-4 recruits SHIP2 to inhibit the PI3K/AKT pathway, maintaining T cells in a hyporesponsive state. Tregs, which express high levels of CTLA-4, use these mechanisms to suppress peripheral immunity and sustain immune tolerance, a process that can be exploited for tumor immune evasion. Created with BioGDP.com [27].

CTLA-4 inhibits T-cell activation primarily by competing with CD28 for binding to the costimulatory ligands CD80/CD86 on antigen-presenting cells (APCs), thereby attenuating CD28-mediated signaling [25, 109]. In addition, CTLA-4 can remove and degrade CD80/CD86 from the APC surface via trans-endocytosis [110]. CTLA-4 also recruits SHIP2 and promotes the dephosphorylation of PI3K, suppressing PI3K/AKT signaling and thereby dampening T-cell metabolic and transcriptional programs to maintain a quiescent state and prevent overactivation (Figure 4) [25]. Beyond conventional T cells, CTLA-4 is critical for regulatory T cells (Tregs), which use CTLA-4-dependent mechanisms to suppress peripheral immune responses, maintain immune tolerance and prevent autoreactivity against normal tissues [111].

Given its negative regulatory function, CTLA-4 is a pivotal mediator of tumor immune evasion. Tumors exploit CTLA-4 pathways to dampen antitumor immunity and escape immune surveillance. For example, Hingorani *et al*. [112] reported markedly increased CTLA-4 expression on T cells from pediatric osteosarcoma patients, suggesting that CTLA-4 may contribute to immune escape in sarcomas.

### CD47

**Figure 5:**
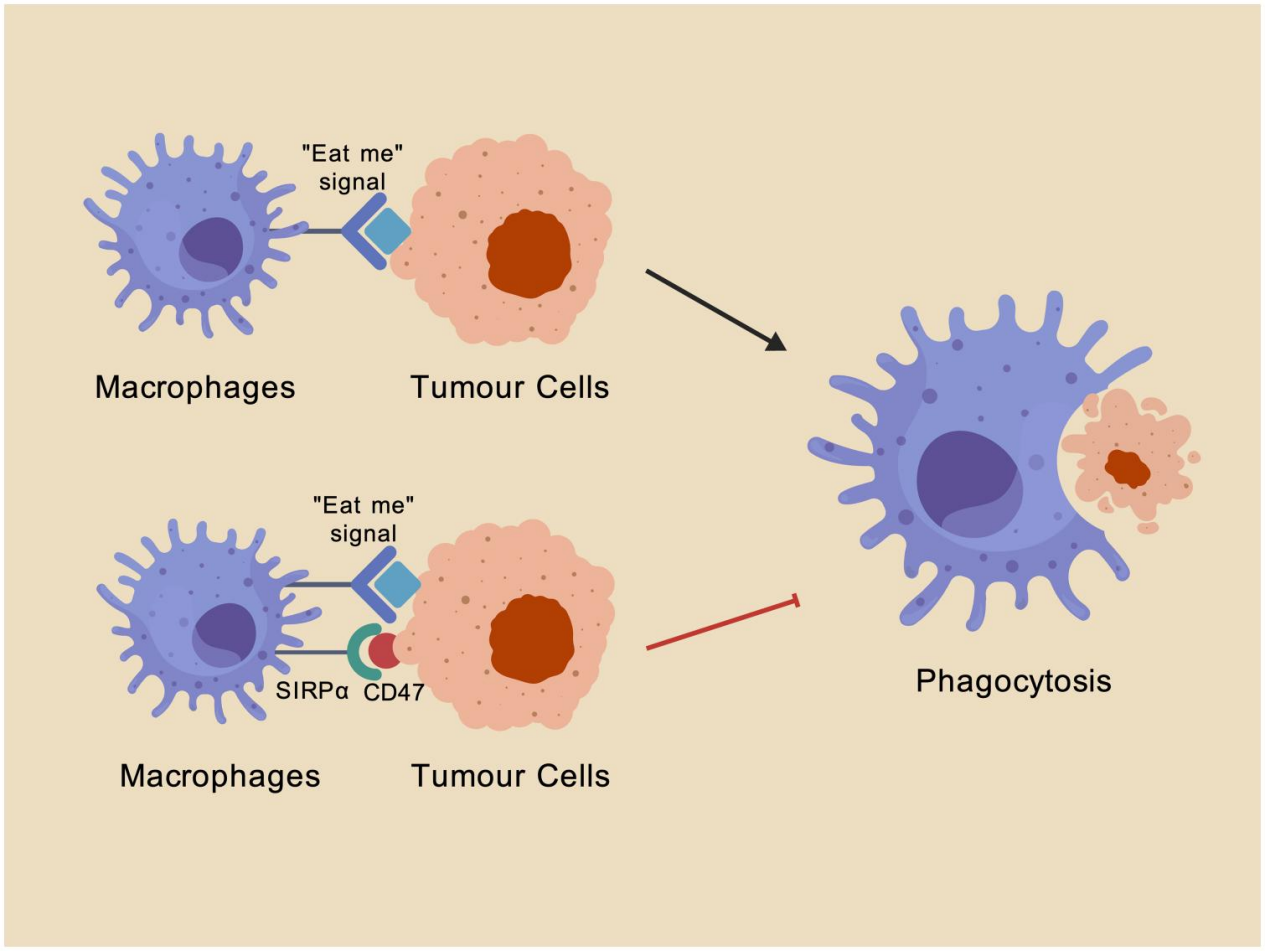
CD47–SIRPα ‘Don’t-eat-me’ signaling pathway mediates macrophage immune evasion in osteosarcoma. Osteosarcoma cells frequently upregulate CD47 as a key inhibitory signal to innate immune surveillance. Binding of CD47 to SIRPα on macrophages activates inhibitory signaling through the cytoplasmic ITIM motifs of SIRPα and leads to the recruitment of SHP-1/SHP-2 phosphatases, which limits cytoskeletal rearrangement and suppresses macrophage phagocytosis. Through this mechanism, tumor cells evade macrophage-mediated clearance and persist within the tumor microenvironment, suggesting that targeting the CD47–SIRPα axis may help restore phagocytic activity and enhance innate antitumor immunity in osteosarcoma. Created with BioGDP.com [27].

CD47 is a transmembrane glycoprotein that is broadly expressed on numerous cell types and is commonly referred to as a ‘don’ t-eat-me’ signal. By binding to signal regulatory protein-α (SIRPα) on macrophages, CD47 inhibits macrophage-mediated phagocytosis (Figure 5) [26]. Oldenborg *et al.* [113] demonstrated that erythrocytes evade macrophage engulfment by engaging with SIRPα via CD47 and reported that SIRPα signaling is mediated through the phosphatase activity of SHP-1. More recently, investigators reported that the CD47/SIRPα axis can also suppress macrophage phagocytic function through SHP2-dependent de-NEDDylation [114]. Tumor cells exploit this pathway by upregulating CD47 expression, thereby restraining macrophage clearance and facilitating tumor growth and dissemination. *In vitro*, Ko *et al.* [115] reported that an anti-CD47 antibody could enhance macrophage phagocytosis of osteosarcoma cells and concurrently inhibit osteosarcoma cell migration. Mechanistically, efforts to elucidate how osteosarcomas upregulate CD47 have indicated that chemotherapy-activated tumor-associated macrophages secrete IL-18, which induces LAT2 expression and augments glutamine and leucine uptake; in turn, this process enhances CD47 expression via the mTORC1/c-Myc pathway, ultimately suppressing macrophage phagocytosis of osteosarcoma cells [76].

In summary, CD47 functions as an immune checkpoint that plays a pivotal role in immune escape in osteosarcoma. Further work is warranted to delineate the determinants of CD47 overexpression in tumors and to define the downstream mechanisms involved in the CD47/SIRPα signaling cascade.

### Immune checkpoint-based immunotherapy

Given the pivotal roles of immune checkpoints such as PD-1, CTLA-4 and CD47 in osteosarcoma immune evasion, inhibitors targeting these pathways have garnered increasing attention in recent years. Many investigators have sought to attenuate tumor immune escape by blocking checkpoint signaling in osteosarcoma. Lussier *et al.* [116] reported that metastatic osteosarcoma commonly exhibits high PD-L1 expression on tumor cells together with PD-1 upregulation on infiltrating cytotoxic T lymphocytes (CTLs); blockade of the PD-1/PD-L1 axis significantly restores CTL function, reduces pulmonary metastasis and prolongs survival.

#### PD-1

In 2018, Zheng *et al.* [117] demonstrated that nivolumab (a PD-1 inhibitor) suppresses osteosarcoma metastasis in mice by promoting the infiltration of CD4^+^ and CD8^+^ T cells and enhancing the cytolytic activity of CD8^+^ T cells within lung metastases. In addition to augmenting T-cell antitumor activity, anti-PD-1 antibodies can also inhibit pulmonary metastasis by activating M1-polarized macrophages while decreasing M2 macrophages or by limiting the infiltration of regulatory T cells (Tregs) in osteosarcoma [54, 118]. Collectively, the results of *in vitro* and *in vivo* studies indicate that PD-1/PD-L1 blockade reshapes multiple immune cell compartments within the tumor microenvironment (TME), thereby restoring antitumor immunity and mitigating immunosuppression.

Clinically, several trials have reported the limited efficacy of PD-1 inhibitors in osteosarcoma and other solid tumors [119, 120]. However, combination strategies appear to enhance antitumor activity. For example, a case report described an advanced, metastatic osteosarcoma patient who achieved a durable remission of 11.7 months following combined therapy with an anti-PD-1 antibody (sintilimab) and the antiangiogenic agent anlotinib [121]. In addition, a real-world study in 2025 revealed that among patients with metastatic osteosarcoma after failure of initial tyrosine kinase inhibitor (TKI) therapy, compared with lenvatinib monotherapy, lenvatinib plus a PD-1 inhibitor significantly prolonged progression-free survival (PFS) (8.6 vs. 4.0 months) [122].

**Figure 6:**
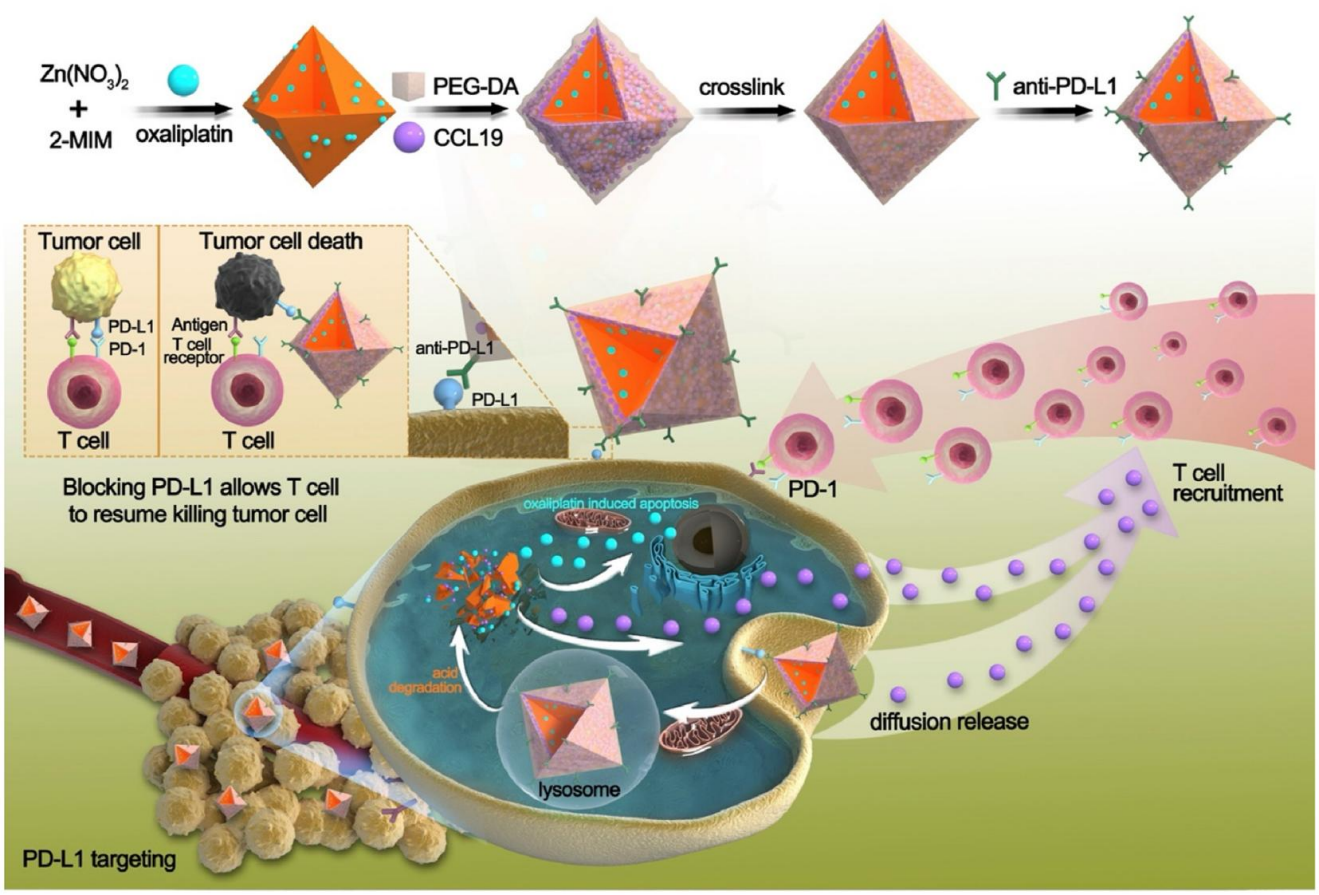
Tumor-targeted stratified MOF system for immune microenvironment modulation in osteosarcoma. A stratified metal-organic framework (MOF) is designed for localized delivery of multiple agents within the osteosarcoma tumor microenvironment. The layered structure enables the spatially controlled release of an anti-PD-L1 antibody, C–C motif chemokine ligand 19 (CCL19) and oxaliplatin. Anti-PD-L1 alleviates immune checkpoint-mediated T-cell inhibition, while CCL19 facilitates the recruitment and infiltration of CD8^+^ T cells into tumor tissue. Oxaliplatin contributes to tumor cell killing and further supports immune activation. By combining immune checkpoint blockade, immune cell recruitment and chemotherapy within a single delivery platform, this system addresses limited T-cell infiltration and enhances local antitumor immune responses in osteosarcoma. Reproduced with permission from Ref. [123].

Li *et al.* [123] proposed a tumor-targeting strategy based on a stratified metal-organic framework (MOF) that loads an anti-PD-L1 antibody, C-C motif chemokine ligand 19 (CCL19), and oxaliplatin into distinct MOF layers. Both the local release of these agents restrained T-cell exhaustion and the recruitment of additional T cells into the TME, thereby overcoming insufficient CD8^+^ T cells infiltration and suppressing osteosarcoma growth and metastasis (Figure 6). This layered MOF-immunotherapy approach offers a new paradigm for managing postoperative bone defects in osteosarcoma and, when integrated with tissue-engineering materials, may simultaneously facilitate defect repair and reduce tumor recurrence.

#### CTLA-4

In 2016, Merchant *et al.* [124] conducted a phase I clinical trial evaluating the safety and antitumor activity of ipilimumab (anti-CTLA-4 antibody) in pediatric patients with advanced solid tumors. Immune-related adverse events (irAEs) were generally manageable, and treatment increased the numbers of activated and circulating T cells without increasing regulatory T cells. Ultimately, 25% of patients with osteosarcoma achieved stable disease following ipilimumab therapy. A more recent study assessed the combination of tremelimumab (a CTLA-4 inhibitor) and durvalumab (a PD-L1 inhibitor) in advanced or metastatic soft-tissue and bone sarcomas. To date, no significant survival benefit has been observed in patients with osteosarcoma; however, investigators have reported the antitumor activity of the combination in advanced or metastatic sarcomas, warranting further evaluation in selected subgroups [125].

#### CD47

Clinical trials of anti-CD47 antibodies specifically in osteosarcoma have not yet been initiated. Nonetheless, experimental studies have demonstrated that CD47 is overexpressed in primary osteosarcoma and that anti-CD47 monoclonal antibodies can block the CD47–SIRPα signaling axis, increase macrophage-mediated phagocytosis of osteosarcoma cells, and suppress spontaneous metastasis [126]. In 2025, in a murine model, Shrestha *et al.* [67] reported that an anti-CD47 antibody could increase the antitumor efficacy of WP1066 (a STAT3 inhibitor) in osteosarcoma, prolonging survival in mice with pulmonary metastases.

In summary, immune checkpoint inhibitors have shown significant value in suppressing tumor immune escape, controlling metastasis, and prolonging survival, providing new opportunities for addressing the clinical challenges of osteosarcoma, including recurrence, therapeutic resistance, and distant metastasis. However, the unique characteristics of bone circulation result in limited efficacy of conventional systemic administration in osteosarcoma. Therefore, the combination of immune checkpoint inhibitors with tissue-engineered materials can achieve spatiotemporally controlled drug release at the tumor site, enhance local immune responses, and simultaneously promote bone defect repair, representing an important developmental direction for precise immunotherapy of osteosarcoma (Table 4).

**Table 4:** Research on immune checkpoint therapy in osteosarcoma.

Drug or materials	Target	Study type	Research results	Time	Country	Ref.
Anti-PD-1 and anti-PD-L1 monoclonal	PD-1/PD-L1 pathway	Preclinical Research	Blockade of PD-1/PD-L1 interactions dramatically improves the function of osteosarcoma-reactive CTLs in vitro and in vivo, and results in decreased tumor burden and increased survival in the K7M2 mouse model of metastatic osteosarcoma.	2015	USA	[116]
Nivolumab	PD-1/PD-L1 pathway	Preclinical Research	Nivolumab inhibits osteosarcoma metastasis in humanized mice by increasing CD4^+^ and CD8^+^ T cells and the cytolytic activity of CD8^+^ T cells in the lung but does not affect primary osteosarcoma growth.	2018	China	[117]
4H2	PD-1/PD-L1 pathway	Preclinical Research	Administration of anti-PD-1 antibody can suppress tumor growth and prolong overall survival, while simultaneously reducing intratumoral regulatory T cells (Tregs) and enhancing tumor-infiltrating lymphocytes within the tumor microenvironment.	2020	Japan	[54]
RMP1-14	PD-1/PD-L1 pathway	Preclinical Research	Anti-PD-1 antibody therapy suppresses pulmonary metastasis of osteosarcoma by blocking oncogenic signaling pathways, enhancing tumor cell apoptosis and reshaping the tumor microenvironment toward increased NK cell and M1 macrophage infiltration with reduced M2 macrophages.	2018	USA	[118]
Sintilimab and anlotinib	PD-1/PD-L1 pathway and VEGFR/FGFR/PDGFR pathway	Case Report	The patient obtained an 11.7-month-sustained remission period, and he also enjoyed better quality of life.	2024	China	[121]
Lenvatinib and camrelizumab, tislelizumab, sintilimab	PD-1/PD-L1 pathway and VEGFR/FGFR pathway	Clinical Research	In patients with metastatic osteosarcoma who experienced failure of initial tyrosine kinase inhibitor (TKI) therapy, lenvatinib combined with a PD-1 inhibitor significantly prolonged PFS compared with lenvatinib monotherapy (8.6 vs. 4.0 months).	2025	China	[122]
Hierarchical Metal-Organic Framework (MOF) loaded with PD-L1 antibody, CCL19 and oxaliplatin	PD-1/PD-L1 pathway	Preclinical Research	It both inhibits T-cell exhaustion and recruits additional T cells into the tumor microenvironment, thereby reversing the insufficiency of CD8⁺ T cells infiltration and suppressing the growth and metastasis of osteosarcoma.	2025	China	[123]
Ipilimumab	CTLA-4 pathway	Clinical Trials	Ipilimumab-related immune adverse events were acceptable, and treatment increased the number of activated and circulating T cells without elevating regulatory T cells; ultimately, 25% of osteosarcoma patients achieved disease stabilization following ipilimumab therapy.	2016	USA	[124]
Tremelimumab and durvalumab	CTLA-4 pathway and PD-1/PD-L1 pathway	Clinical Trials	The combination of tremelimumab (a CTLA-4 inhibitor) and durvalumab (a PD-L1 inhibitor) showed no significant survival benefit in osteosarcoma but demonstrated activity in advanced or metastatic sarcomas, warranting further evaluation in specific patient subgroups.	2022	USA	[125]
B6H12 and Ab400	CD47-SIRPα pathway	Preclinical Research	Anti-CD47 monoclonal antibody blocks the CD47–SIRPα signaling pathway, thereby enhancing macrophage-mediated phagocytosis of osteosarcoma cells and suppressing spontaneous metastasis of osteosarcoma.	2015	China	[126]
MIAP410	CD47-SIRPα pathway	Preclinical Research	Anti-CD47 antibody enhances the antitumor activity of WP1066 (a STAT3 inhibitor) in a murine osteosarcoma model and prolongs the survival of mice witumor relapse and worsen.	2025	USA	[67]

## Challenges in postoperative bone defect repair after osteosarcoma and immuno-tissue engineering strategies

### Conventional tissue-engineering biomaterials for postoperative bone defect repair in osteosarcoma: advantages and limitations

Although immunotherapeutic approaches have shown promise in alleviating the immunosuppressive state of the osteosarcoma tumor microenvironment (OS-TME) and reducing the risk of postoperative recurrence, conventional tissue-engineering biomaterials remain indispensable for providing structural support and promoting osteogenesis in postoperative bone defect repair. These materials, including calcium phosphate-based ceramics, polymeric biomaterials, metallic biomaterials and composite systems, have been widely applied to mimic the bone extracellular matrix, facilitate cell adhesion and accelerate bone regeneration. However, in the context of osteosarcoma, their application is constrained by a lack of intrinsic antitumor activity. Moreover, the release of growth-promoting factors or ions from these materials may inadvertently bias the local immune microenvironment toward an anti-inflammatory, tumor-permissive state, thereby supporting the survival and proliferation of residual tumor cells. To clarify both the regenerative advantages and oncological limitations of conventional tissue-engineering biomaterials, representative material systems are summarized with emphasis on their strengths, weaknesses, safety profiles, long-term biocompatibility, translational relevance and mechanical performance. In particular, the range of Young’s modulus is highlighted as a key parameter for evaluating load-bearing suitability, with the aim of elucidating how these materials address the challenges of bone defect repair while revealing their application potential and inherent constraints.

Calcium phosphate-based ceramics, such as hydroxyapatite (HA), β-tricalcium phosphate (β-TCP) and biphasic calcium phosphate (BCP), are widely favored due to their close resemblance to native bone mineral. These materials exhibit excellent biocompatibility and osteoconductivity, supporting osseointegration and osteogenesis through ion release. Nevertheless, their intrinsic brittleness and mismatched degradation kinetics restrict broader application, with Young’s moduli typically ranging from 1 to 8 GPa, rendering them more suitable for cancellous bone repair [127–132]. Polymeric biomaterials, including PLGA, PLA and PCL, emulate the organic phase of bone and offer tunable degradation behavior and cellular support; however, their limited bioactivity and the potential inflammatory responses induced by acidic degradation byproducts constrain their application primarily to drug delivery or nonload-bearing scenarios, with Young’s moduli generally ranging from 0.075 to 2.46 GPa [133–137]. Nondegradable metallic biomaterials, such as titanium alloys and tantalum, provide superior mechanical strength and reliable osseointegration, exhibiting Young’s moduli of approximately 110–185 GPa, and thus, being well suited for load-bearing reconstruction. Their nondegradable nature, however, may lead to stress-shielding effects, which can be partially mitigated through porous or architecturally optimized designs [138, 139]. In contrast, biodegradable metallic biomaterials, including magnesium- and zinc-based alloys, possess mechanical properties closer to those of native bone and can promote osteogenesis, with Young’s moduli ranging from approximately 0.18–2.27 GPa. Nonetheless, their degradation products may introduce potential oncological risks; for example, magnesium and zinc ions, while enhancing osteogenesis and angiogenesis, have also been reported to exacerbate tumor cell migration and neovascularization under certain pathological conditions [140–143]. Composite biomaterials integrate the advantages of multiple material classes, enabling improvements in bioactivity, mechanical performance and degradation behavior while supporting porous architectures and customized designs, with Young’s moduli typically ranging from 0.81 to 4.8 GPa [144–146]. Overall, although conventional biomaterials have matured in providing osteoconductive support, tissue integration and mechanical stability, their lack of active antitumor regulatory capacity remains a critical limitation in addressing recurrence risk following osteosarcoma resection (Table 5).

In summary, repair of bone defects following osteosarcoma resection remains a major clinical challenge. Conventional tissue engineering relies on the triad of seed stem cells, cytokines/growth factors and scaffold materials to promote bone regeneration by stimulating the proliferation and differentiation of osteogenic cells [147]. However, these stimulatory cues may also activate residual osteosarcoma cells, leading to tumor recurrence or metastasis and thereby substantially increasing the risk of unfavorable outcomes in patients [147, 148]. Without tissue engineering approaches, large segmental defects are difficult to repair and may result in functional limitations or even disability; however, the straightforward application of conventional tissue engineering may, conversely, trigger tumor relapse and worsen prognosis [149, 150].

**Table 5:** Advantages and limitations of conventional tissue-engineering biomaterials in osteosarcoma-associated bone defect repair.

Category	Subtype	Advantages	Disadvantages	Safety	Long-term biocompatibility	Translational relevance	Mechanical properties	Ref.
Calcium Phosphate-Based Ceramics	(a) Hydroxyapatite (HA)	Excellent biocompatibility and osteoconductivity; forms direct bond with bone via apatite layer; enhances osseointegration.	Brittleness; low fracture toughness; porosity reduces strength.	Low antigenicity; safe; no disease transmission.	Supports bone formation; minimal immunoreactions; nanoparticles may cause cytotoxicity.	Used in orthopedic/dental; 3D-printed for defects.	pore size: 200–400 μm; Young’s modulus ≈8 GPa; compressive yield strength ≈5 MPa	[127]
	(b) β-Tricalcium Phosphate (β-TCP)	Higher solubility; degrades faster than HA; releases ions supporting osteoblast activity.	Rapid degradation may cause instability; fibrous encapsulation.	Degradation products integrate into metabolism; safe.	Minimal inflammation; integrates into metabolism.	β-TCP+MSCs reduce radiolucency; used in defects.	Young’s modulus: 2–4 GPa; compressive strength: 20–30 MPa	[128, 129]
	(c) Biphasic Calcium Phosphate (HA + TCP)	Tunable degradation; balances stability and resorption for bone ingrowth.	Brittleness; variable degradation.	Low inflammatory responses; safe.	Supports bone formation; low immunoreactions.	3D-printed for defects; enhances bone quality.	Balanced mechanical performance; Young’s modulus: 1–4 GPa; compressive strength: 6.20 ± 0.81 MPa	[130–132]
Bioactive Glass	Bioactive Glass	Releases ions stimulating osteogenesis, angiogenesis; enhances gene expression for bone/vascularization.	Rapid degradation in particles causes instability/inflammation; limited strength.	Biocompatible; low inflammation.	Forms apatite layers; supports integration.	Used in tibial defects; combined with polymers.	Average pore size: 106 μm; compressive strength ≈ 3.7 MPa	[151]
Polymeric Biomaterials	(a) PLGA (Poly(lactic-co-glycolic acid))	Tunable degradation; good biocompatibility; mimics bone’s organic phase; supports cell activity when modified.	Poor bioactivity; acidic byproducts cause inflammation; strength below cortical bone.	Degrades to non-toxic products; low toxicity.	Modifications enhance integration.	3D-printed/electrospun; drug delivery in osteosarcoma.	Tensile strength: 45–52.7 MPa; Young’s modulus: 2.28–2.46 GPa	[133]
	(b) PLA (Polylactic acid)	Good biocompatibility; supports cell activity when modified.	Poor bioactivity; acidic byproducts; strength-degradation trade-offs.	Low toxicity; safe.	Enhances integration with modifications.	Composites for delivery.	Compressive strength: 0.25 -8.22 Mpa; Young’s modulus: 0.075 GPa	[134, 135]
	(c) PCL (Polycaprolactone)	Tunable degradation; mimics organic phase; supports cell activity; blends increase bone volume.	Poor bioactivity; acidic byproducts; strength below cortical.	Low toxicity; safe but acidic byproducts hinder growth.	Modifications improve integration.	Blends increase bone volume; substitutes.	compressive strength: 0.01–16.18 Mpa; Young’s modulus: 0.44–0.532 GPa	[136, 137]
Metallic Biomaterials	(a) Titanium and Titanium Alloys	High strength; corrosion resistance; osseointegration with treatments.	Nonbiodegradable; stress shielding; ion release; infections.	Biocompatible; wear particles inflame; modifications help.	Good; particles may inflame; modifications aid.	Dominant in implants; 3D-printed for defects.	Bulk Ti: Young’s modulus ≈ 110 GPa; porous Ti (porosity 47.75–49.97%): effective Young’s modulus: 7.50–10.75 GPa	[138, 139]
	(b) Tantalum	High strength; porosity for ingrowth.	Nonbiodegradable; stress shielding; ion release.	Biocompatible; modifications reduce risks.	Supports integration.	Porous implants; coatings enhance.	Bulk Ta: Young’s modulus ≈ 185 GPa; porous Ta (porosity 60–80%): effective Young’s modulus: 0.35–2.3 GPa	[138]
	(c) Magnesium Alloys	Biodegradable; properties close to bone; promotes osteogenesis/angiogenesis.	Rapid degradation; hydrogen gas/inflammation; integrity loss.	Safe; hydrogen manageable; no adverse reactions.	Biodegradable products integrate; minimal inflammation.	Orthopedic implants; trials for fixation/regeneration.	Yield strength: 270 ± 9.29 MPa; tensile strength: 330 ± 15.8 MPa; degradation rate: 0.35 ± 0.02 mm·year⁻¹ Young’s modulus: 0.18-2.27 GPa	[140, 141]
	(d) Zinc Alloys	Biodegradable; moderate corrosion; promotes bone formation; tunable properties.	Cytotoxicity from high Zn release; optimization needed.	High biocompatibility; low toxicity with alloying.	Supports integration; enhances osteoconductivity.	Orthopedic implants; membranes; superior bone formation.	Yield strength: 198.7 MPa; tensile strength: 217.6 Mpa Young’s modulus: 1.17 ± 0.11 GPa	[142, 143]
Composite Biomaterials	(a) Combinations like Polymer + Ceramic or Metal + Bioactive Coating	Enhanced bioactivity/mechanics/degradation; porosity for ingrowth.	Complexity; weak interfaces; solvent toxicity.	Improved; low risks.	Promotes differentiation/integration.	Limb reconstruction; 3D-printed for defects.	Compressive strength: 42.07–59.81 MPa; Young’s modulus: 0.81–0.91 GPa	[144]
	(b) Designed to Mimic Natural Bone (Strength + Bioactivity)	Tailored to mimic bone; enhanced bioactivity/mechanics.	Design complexity; interface weaknesses.	Enhanced; low ion risks.	Supports bone mimicry.	Advanced reconstruction.	Total porosity: 34.5 ± 5.6 %; Young’s modulus: 4.8 ± 0.1 GPa; compressive strength: 21.9 ± 6.2 MPa	[145, 146]

### Immune microenvironment modulation as an independent ‘fourth element’ in tissue engineering

Effective treatment of osteosarcoma requires not only the reconstruction of bone defects but also the suppression of tumor recurrence and metastasis. Conventional bone tissue-engineering strategies primarily focus on optimizing the integration of three core elements—seed cells, scaffold materials and growth factors. Despite notable advances in promoting bone regeneration, clinical evidence has increasingly indicated an elevated risk of postoperative tumor recurrence associated with these approaches [152]. Retrospective clinical cohort studies have reported local recurrence rates of up to 27–33% following postoperative bone defect reconstruction, largely attributable to residual tumor cells escaping immune surveillance [153]. Animal studies further demonstrate that growth factors such as vascular endothelial growth factor (VEGF), while enhancing vascularization, may inadvertently stimulate tumor angiogenesis [154], and that bone morphogenetic protein-2 (BMP-2) can significantly promote osteosarcoma cell proliferation, migration and invasiveness [155]. In addition, magnesium-containing scaffolds have been shown to induce macrophage polarization toward an M2 phenotype, potentially facilitating osteosarcoma progression [156]. Collectively, these findings indicate that traditional tissue-engineering strategies often overlook the regulation of the host immune system, which not only limits their capacity to suppress tumor recurrence and metastasis but may, in some cases, exacerbate disease progression [157].

In recent years, the central role of the immune system in bone tissue engineering has gained increasing recognition. The immune microenvironment, composed of diverse immune cells and mediators, exerts dual effects on bone regeneration as well as tumor recurrence and metastasis. Macrophages exemplify this dichotomy: M1-polarized macrophages mediate inflammatory responses that facilitate the clearance of residual tumor cells, whereas M2-polarized macrophages promote tissue repair and osteogenic differentiation but are also associated with increased risks of tumor recurrence and metastasis [158]. Notably, the osteosarcoma immune microenvironment is typically characterized by an immunosuppressive state, including elevated expression of IL-10 and TGF-β, which increases the likelihood of immune evasion by tumor cells [50]. Preclinical evidence further suggests that the absence of immune modulation amplifies the adverse effects of the traditional three elements. Scaffold-induced foreign body responses may aggravate chronic inflammation, while growth factor-mediated signaling can be co-opted by tumor cells to promote therapeutic resistance and recurrence [157]. Consequently, although the classical triad provides a foundational framework for bone regeneration, its inability to actively regulate the immune microenvironment precludes the achievement of a balanced outcome between antitumor control (recurrence and metastasis) and tissue regeneration.

To address this challenge, the traditional three elements of tissue engineering require redesign by introducing strategies that modulate the immune microenvironment, thereby integrating immune regulation as a ‘fourth element’ alongside seed cells, scaffolds and bioactive factors. This integrated paradigm seeks to enhance immune recognition and clearance of residual osteosarcoma cells while simultaneously supporting bone regeneration, achieving balanced antitumor effects and reducing the risk of recurrence. The following sections examine such integrated approaches, encompassing strategies such as temporally programmed release, multimaterial integration and spatiotemporal specificity, among others.

### Sequential release strategy: staging antitumor and proregenerative components via biomaterial design

Early burst release suppresses tumor growth, whereas subsequent sustained release promotes bone repair. This approach establishes a controlled-degradation system that aligns the release sequence with clinical priorities and reduces the risk of concurrently affecting both tumor cells and osteogenic cells. In the postoperative osteosarcoma setting, the initial release phase eliminates residual malignant cells; the later phase activates osteogenic signaling, thereby lowering the recurrence risk and accelerating the healing of bone defects.

Within metallic systems, Mg implants show broad potential as reconstruction materials for postresection defects in osteosarcoma. Zhang *et al.* [159] reported that Mg implants not only promote bone regeneration through the release of Mg^2+^ but also generate an alkaline microenvironment during degradation that provides antibacterial and antitumor effects. However, the release of Mg^2+^ also increases the likelihood of M2 macrophage polarization and tumor cell migration and proliferation. Therefore, effectively curbing the early burst release from Mg-based materials during the initial postoperative period is a key challenge; if achieved, such materials could exhibit substantial promise in postoperative reconstruction.

**Figure 7:**
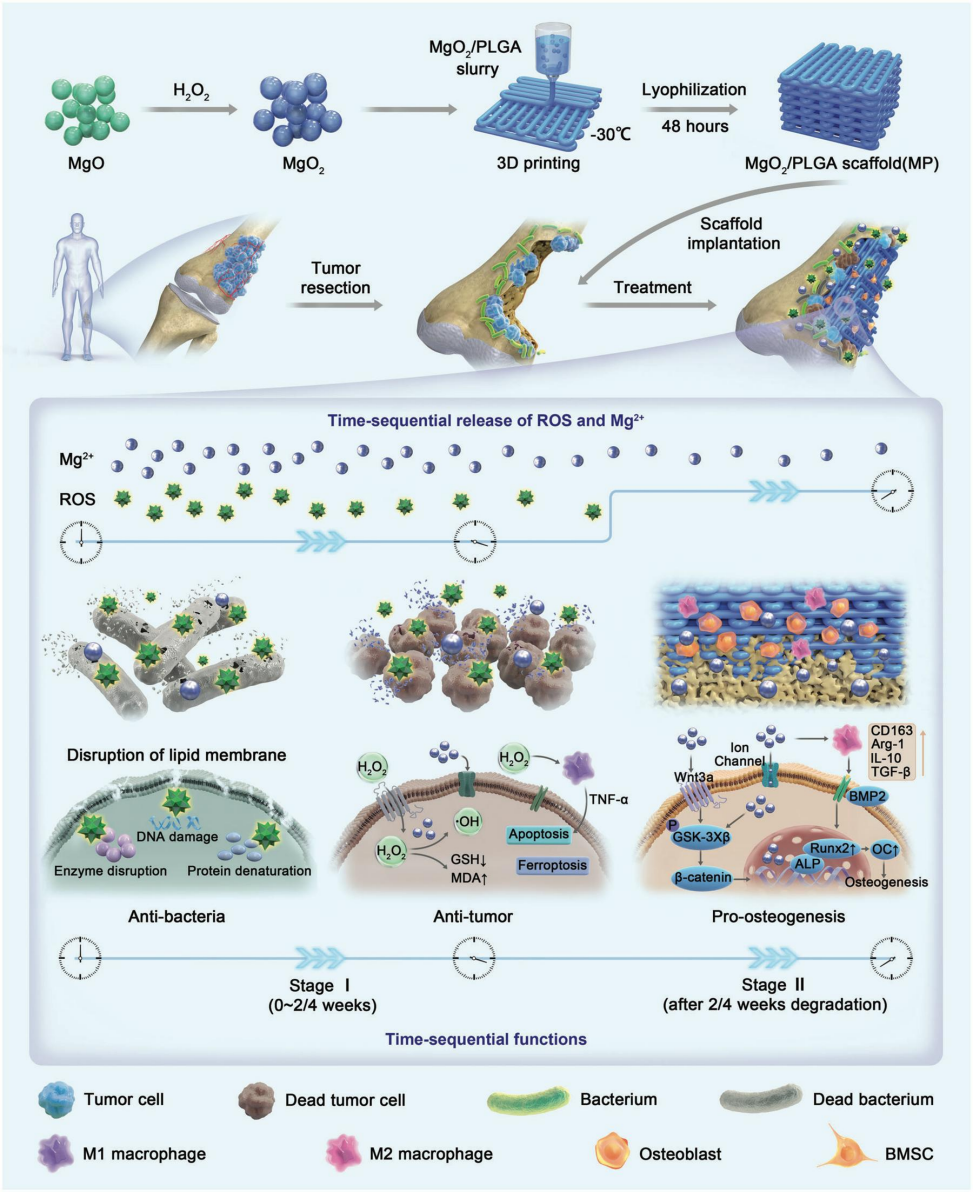
Temporally programmable release integrates immunity and regeneration (exemplified by an MgO_2_/PLGA nanocomposite scaffold). This figure shows a time-programmed strategy using MgO_2_/PLGA nanocomposite scaffolds to promote both immune modulation and bone repair after osteosarcoma surgery. In the early phase (within the first 3 weeks), the rapid release of H_2_O_2_ activates chemodynamic therapy (CDT), inducing tumor cell death and polarizing M1 macrophages to target any remaining osteosarcoma cells. In the later stages, sustained Mg^2+^ release activates the Wnt3a/GSK-3β/β-catenin pathway, which helps mesenchymal stem cells (MSCs) differentiate into osteoblasts and supports angiogenesis. Alongside this, M2 macrophage polarization creates an environment conducive to bone healing. This staged approach effectively combines immune activation to suppress the tumor early on, followed by a regenerative immune response to foster bone repair, offering a promising strategy for enhancing both tumor control and bone regeneration in osteosarcoma patients. Reproduced with permission from Ref. [160].

To address this challenge, Li *et al.* [160] used low-temperature 3D printing to fabricate a nanocomposite scaffold (magnesium peroxide/poly(lactic-co-glycolic acid), MgO_2_/PLGA). The material released hydrogen peroxide during the first 3 weeks and then continuously released Mg^2+^ for the subsequent 12 weeks, enabling temporally ordered, controllable delivery of reactive oxygen species and magnesium ions. Liberated H_2_O_2_ initiates chemodynamic therapy, inducing tumor cell apoptosis and ferroptosis and, via M1 macrophage polarization, establishes an anticancer immune microenvironment. In turn, the sustained Mg^2+^ release activates the Wnt3a/GSK-3β/β-catenin signaling pathway to promote osteogenic differentiation of bone marrow mesenchymal stem cells while fostering a pro-osteogenic immune milieu through M2 macrophage polarization, thereby synergistically enhancing bone repair (Figure 7).

The core advantage of this strategy lies in its programmable temporal control: by sequentially releasing distinct components to remodel the tumor microenvironment (TME), it aims to intensify antitumor activity in the early phase and facilitate bone repair in the late phase. However, a key challenge remains—how to define, in a patient-specific manner, the optimal duration window for the early antitumor phase.

### Multimaterial integration strategies: consolidating diverse bioactivities

Unlike traditional single-material approaches that rely on a ‘single-point function’, multimaterial integration leverages the complementarity and synergy of heterogeneous phases to achieve, within a single construct, concurrent mechanical matching, controllable degradation and enhanced bioactivity. The core goal is to replace trade-offs with system-level integration, offering a generalizable route for one-stop reconstruction in the complex postoperative microenvironment following osteosarcoma resection.

**Figure 8:**
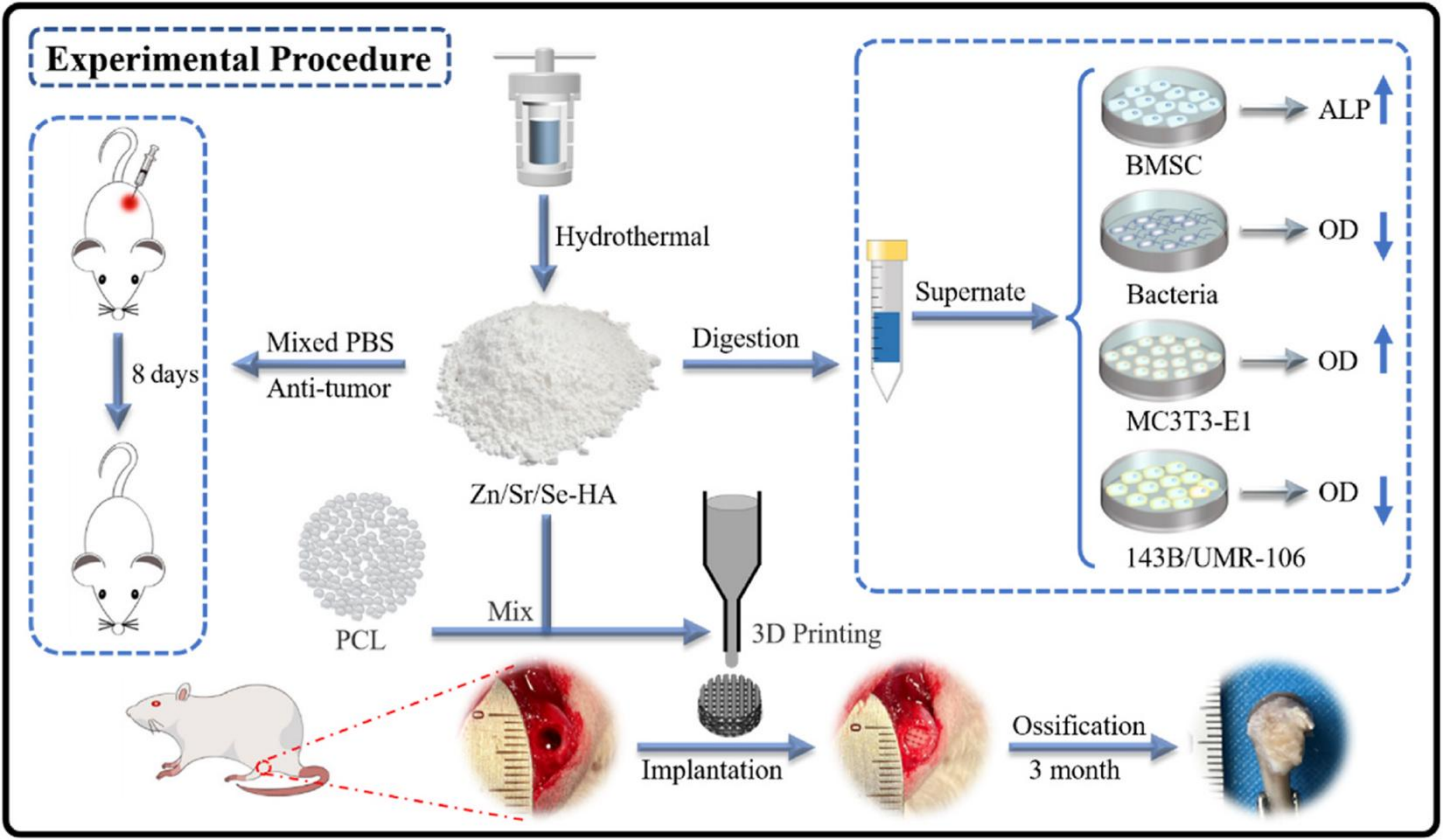
Synergistic antitumor and osteogenic effects of a Se/Sr/Zn-doped HA–PCL composite scaffold. A composite scaffold composed of hydroxyapatite (HA) and polycaprolactone (PCL) is doped with selenium (Se), strontium (Sr) and zinc (Zn) to achieve combined antitumor and pro-osteogenic functions. Selenium primarily contributes to tumor suppression, whereas strontium and zinc support osteogenic differentiation and bone formation. The incorporation of multiple bioactive ions within a single scaffold allows simultaneous regulation of tumor inhibition and bone regeneration, providing a material-based strategy for osteosarcoma-associated bone defect repair. Reproduced with permission from Ref. [161].

For example, Huang *et al.* [161] fabricated 3D-printed composite scaffolds comprising Se/Sr/Zn-doped hydroxyapatite and polycaprolactone (Se/Sr/Zn-HA–PCLs) for repairing postresection bone defects in osteosarcoma. The results demonstrated that selenite (${\text{SeO}}_{3}^{2-}$) has pronounced antitumor activity; moreover, ${\text{SeO}}_{3}^{2-}$, Sr^2+^ and Zn^2+^ each enhances osteogenic differentiation. The resulting Se/Sr/Zn-HA–PCLs, thus, have multiple synergistic effects and confer dual functions of tumor suppression and promotion of bone regeneration (Figure 8).

**Figure 9:**
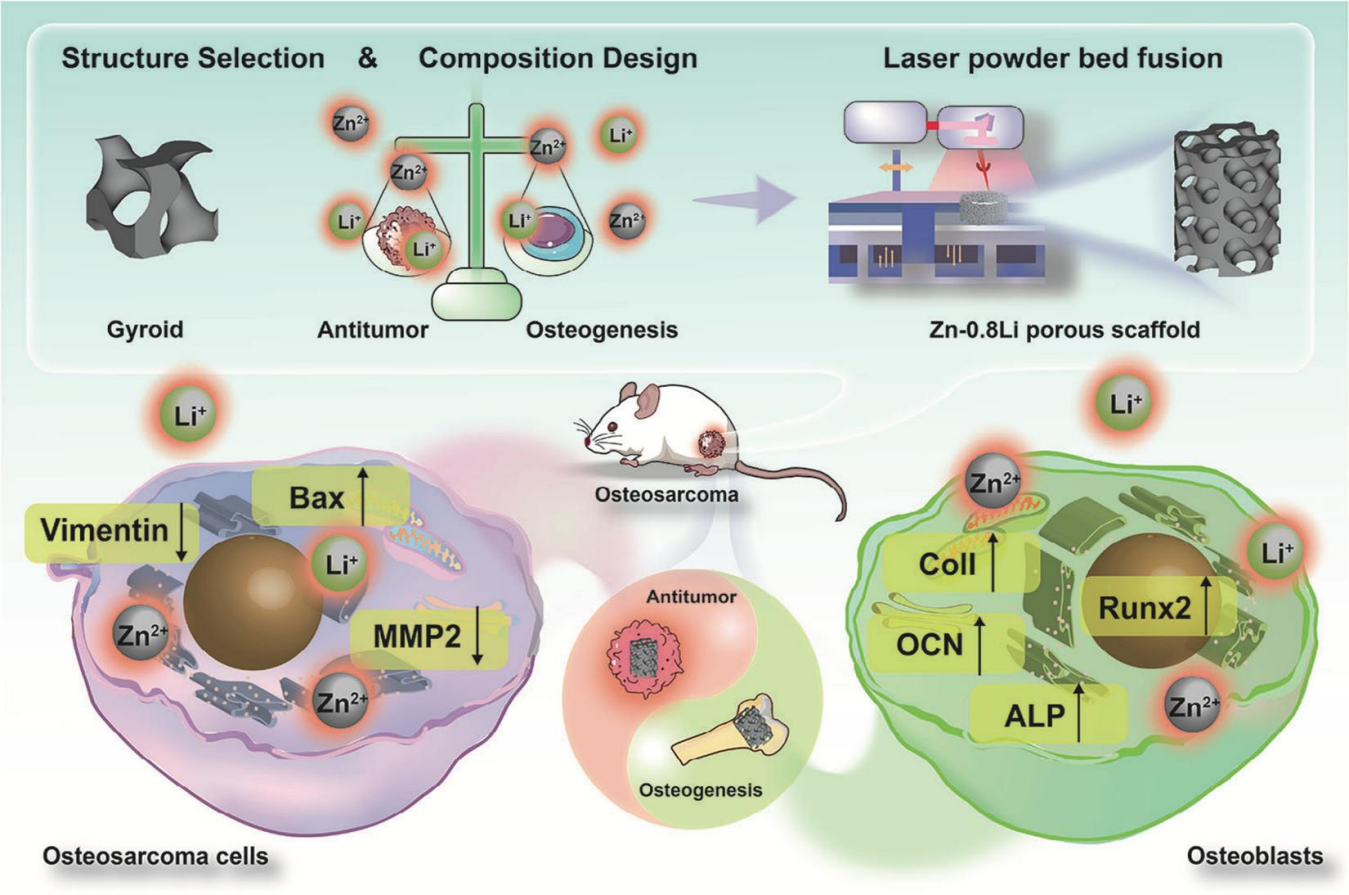
Composition optimization and dual effects of Zn–Li porous alloy scaffolds. Among biodegradable, 3D-printed Zn–Li porous scaffolds, the Zn–0.8Li composition results in the best performance: released Zn^2+^ suppresses osteosarcoma cell proliferation/migration and promotes apoptosis, whereas Li^+^ primarily drives osteogenic phenotypes (e.g. ALP activity and mineralization) and new bone formation, achieving concurrent tumor inhibition and bone regeneration. This scaffold design achieves a dual therapeutic effect, combining tumor inhibition with bone regeneration in a single platform. By carefully tuning the alloy composition, this approach offers a promising solution for treating osteosarcoma and promoting bone healing. Reproduced with permission from Ref. [162].

Lu *et al.* [162] 3D-printed degradable zinc–lithium alloy porous scaffolds and subsequently reported that the Zn–0.8Li scaffold exhibited optimal antitumor and osteogenic properties. The controlled release of Zn^2+^ inhibited proliferation, promoted apoptosis and reduced the migration of osteosarcoma cells, whereas Li^+^ primarily drove osteogenic differentiation and new bone formation (Figure 9).

The advantage of these scaffolds lies in their compositional tunability (e.g. Zn–0.8Li), which enables simultaneous antitumor activity and bone-regenerative function. However, their potential toxicity to healthy tissues remains unclear; future studies could integrate novel bioactive materials to further optimize their performance.

### Spatiotemporally specific strategies: materials that respond precisely within the tumor microenvironment

Spatiotemporally specific strategies involve the engineering materials to respond to features of the tumor microenvironment (TME)—such as acidity, elevated glutathione (GSH) or exogenous stimuli (e.g. photothermal irradiation)—to enable on-site activation while sparing healthy tissues. This approach achieves precise tumor ablation alongside controlled bone repair. By leveraging TME hallmarks (acidic pH, hypoxia and high GSH levels) to trigger material degradation or functional switching, these systems achieve the dual effects of tumor-specific cytotoxicity and osteogenic activity.

**Figure 10:**
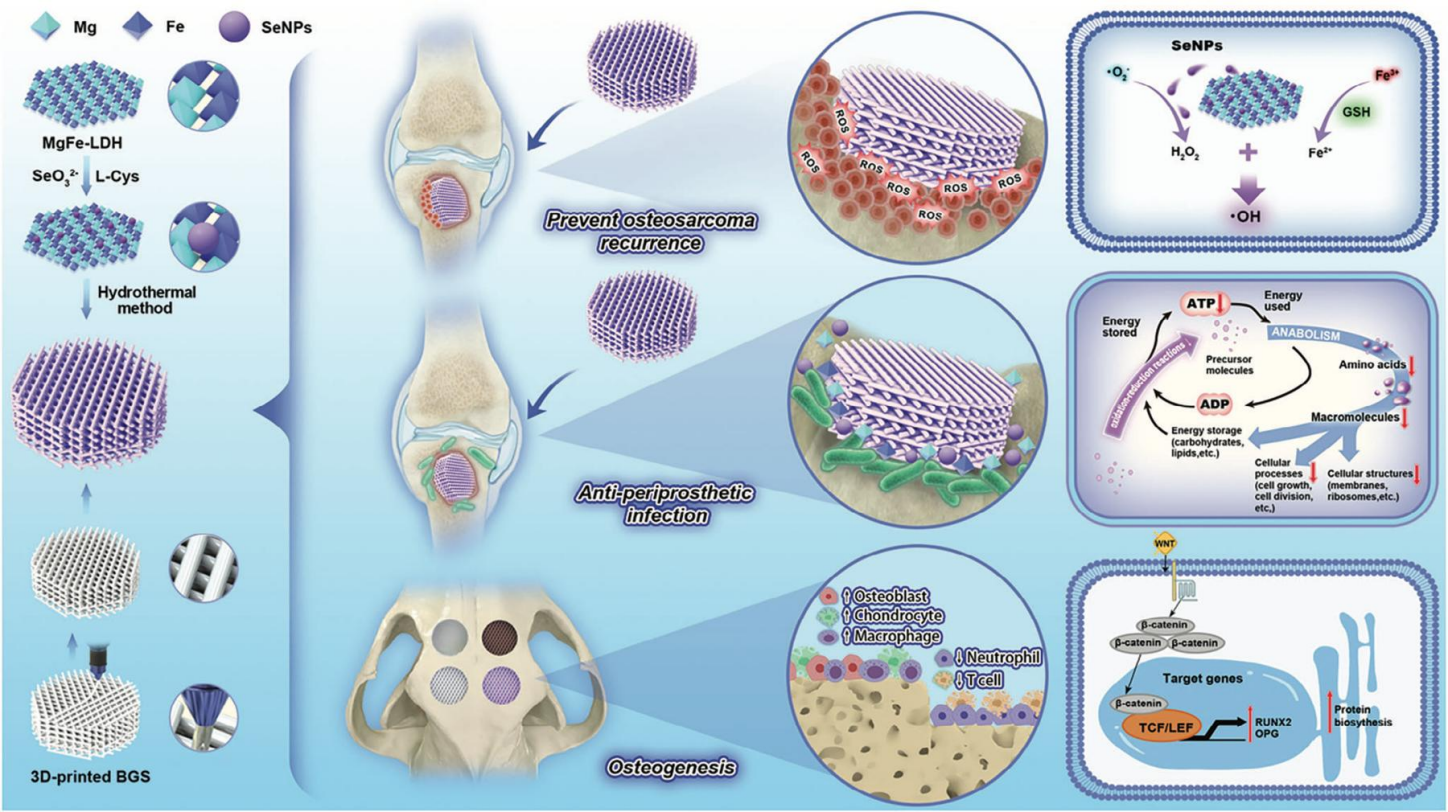
TME-responsive Fenton amplification with concurrent osteogenesis: SeNPs@MgFe-LDH-functionalized bioactive glass scaffold. In the weakly acidic, high-GSH tumor microenvironment, Fe³^+^ is reduced to Fe^2+^ to trigger a (Fenton-like) reaction, locally amplifying ROS to eradicate residual tumor cells. Moreover, the sustained release of Mg^2+^/Fe³^+^/Se activates Wnt-β-catenin signaling, promoting osteogenic differentiation and bone regeneration—thus, achieving tumor-specific death during tissue repair. This dual mechanism makes the scaffold an effective tool for both tumor elimination and bone repair. Reproduced with permission from Ref. [163].

Bian *et al.* [163] developed a selenium-nanoparticle/magnesium–iron layered double hydroxide-functionalized bioactive glass scaffold (SeNPs@MgFe-LDH). In the weakly acidic and GSH-rich TME, Fe³^+^ is reduced to Fe^2+^, initiating a Fenton reaction that locally amplifies reactive oxygen species (ROS) to eliminate residual tumor cells. Concurrently, the sustained release of Mg^2+^/Fe³^+^/Se activates the Wnt-β-catenin pathway to promote osteogenesis (Figure 10).

**Figure 11:**
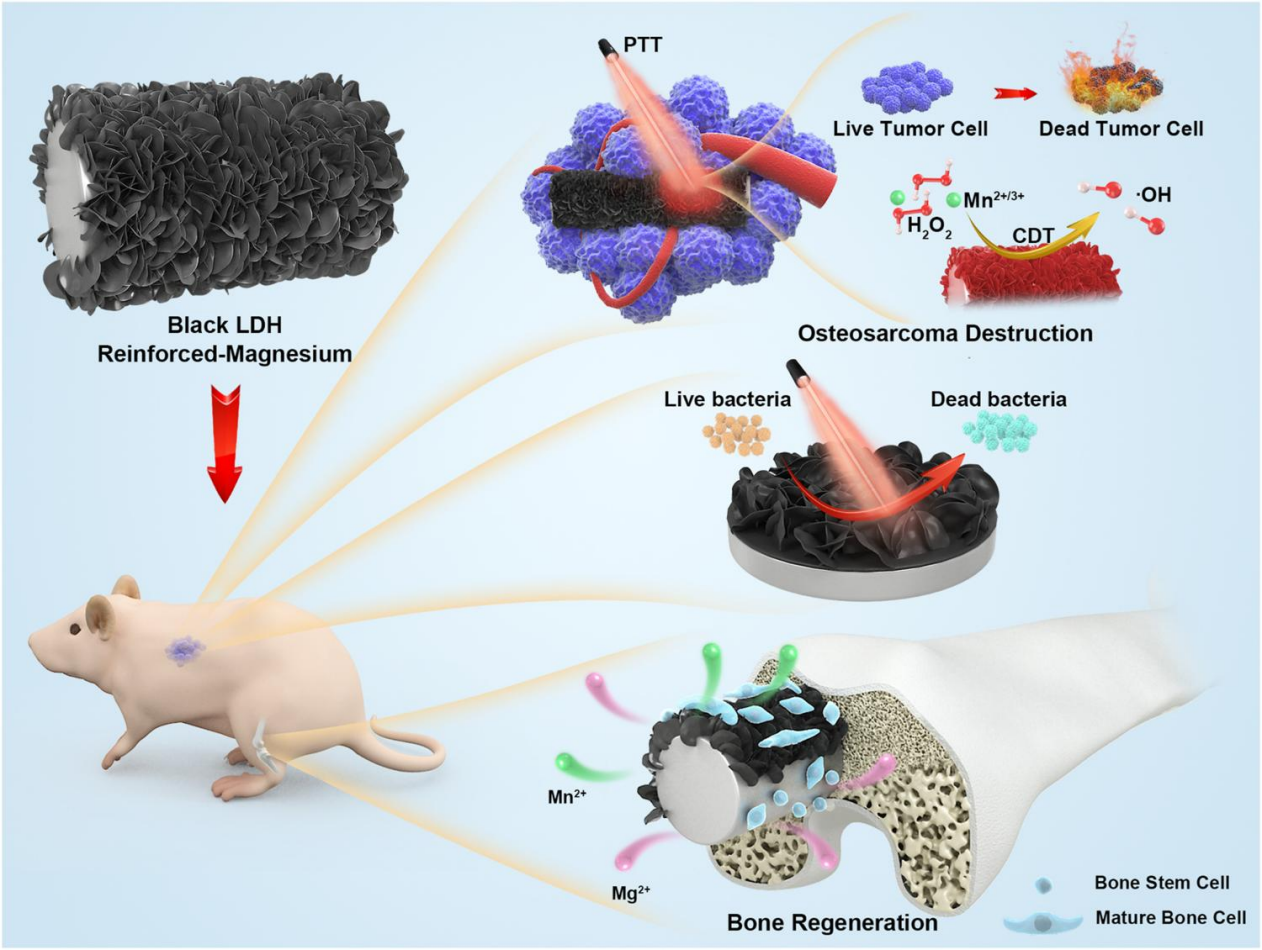
Near-infrared-triggered photothermal-chemodynamic synergy with pro-osteogenic effects: Mn-LDH-coated magnesium alloy. Under NIR irradiation, the black Mn-LDH coating produces photothermal therapy (PTT) and catalyzes a Fenton-like reaction to amplify ROS, thereby efficiently suppressing osteosarcoma. Moreover, the magnesium-alloy substrate, together with the sustained release of Mg^2+^/Mn^2+^, enhances osteogenic cell adhesion, ALP activity and mineralization, accelerating postoperative bone regeneration. This dual mechanism of tumor suppression and bone repair provides a promising approach for postoperative osteosarcoma treatment. Reproduced with permission from Ref. [164].

In addition, Zhang *et al.* [164] constructed a black Mn-LDH coating on degradable magnesium alloys. Under near-infrared irradiation, the platform achieves synergistic photothermal-chemodynamic (Fenton-like) therapy to intensify the ROS-mediated suppression of osteosarcoma. In parallel, the controlled release of Mg^2+^/Mn^2+^ coupled with a bioactive interface enhances osteoblast adhesion, alkaline phosphatase (ALP) activity and mineralization, thereby accelerating bone regeneration (Figure 11).

These materials offer precision and safety: activation by TME cues or external photothermal stimuli confines ROS generation to the tumor site, minimizing off-target injury and systemic toxicity. The remaining challenges include optimizing response thresholds to accommodate inter- and intratumoral heterogeneity in the TME and improving the clinical practicality of external stimulus delivery. Looking ahead, integrating nanotechnology with image guidance is expected to further refine spatiotemporal control and advance precision therapy.

### Synergistic modulation of immune checkpoint blockade with biomaterials

Integrating immune checkpoint blockade (ICB) with biomaterials offers a new avenue to address both bone repair and tumor recurrence. Tumor cells frequently upregulate checkpoint axes such as PD-1/PD-L1 to evade immune surveillance. ICB interrupts these pathways to reactivate T-cell-mediated antitumor immunity, while biomaterial integration enables localized delivery, sustained release and microenvironmental modulation, thereby enhancing therapeutic efficacy and promoting bone regeneration.

**Figure 12:**
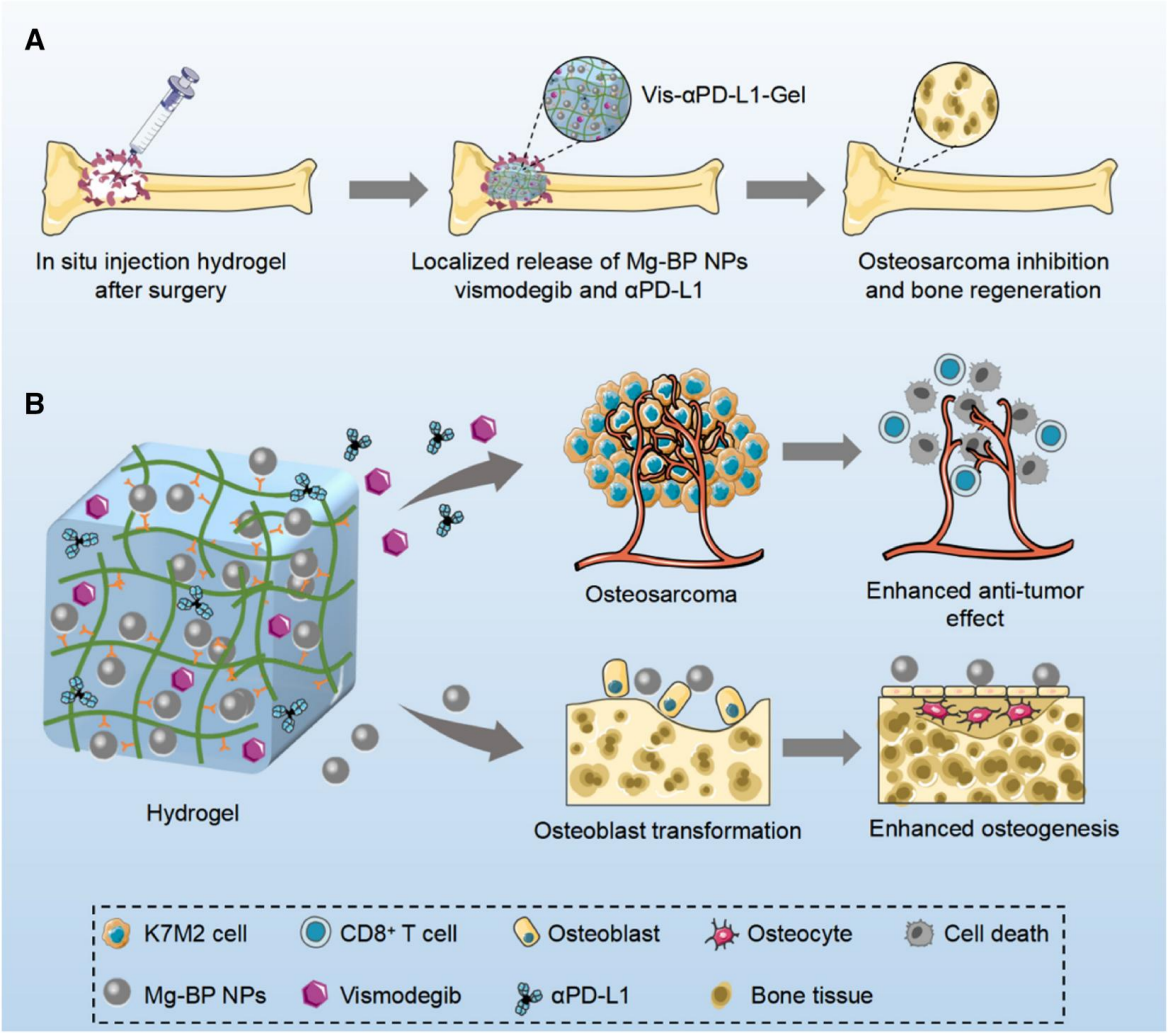
Schematic illustration of bioactive nanocomposite hydrogel for enhanced bone tumor immunotherapy and bone regeneration. (A) The nanocomposite hydrogel is fabricated using bisphosphonate-modified hyaluronic acid (HA-BP) and self-assembled magnesium (Mg)-bisphosphonate (BP) nanoparticles (Mg-BP NPs). (B) At the resection bed, the hydrogel codelivers αPD-L1 and the Hedgehog pathway inhibitor vismodegib while providing sustained Mg^2+^ release; this process reverses T-cell exhaustion and augments antitumor immunity to prevent recurrence and concurrently promotes osteogenic differentiation and bone repair—achieving on-site synergy between immunotherapy and tissue engineering. This approach effectively combines immune checkpoint blockade and bone repair, offering a promising solution for both tumor suppression and bone regeneration after osteosarcoma surgery. Reproduced with permission from Ref. [165].

In 2025, Chu *et al.* [165] proposed a nanocomposite hydrogel coencapsulating an anti-PD-L1 antibody (αPD-L1), a Hedgehog pathway inhibitor (vismodegib) and magnesium ions (Mg^2+^). This platform codelivers αPD-L1 and vismodegib and provides prolonged Mg^2+^ release, achieving sustained immune potentiation at the postoperative resection site to suppress osteosarcoma recurrence while simultaneously promoting osteogenic differentiation and bone regeneration (Figure 12).

This approach leverages the capacity of the hydrogel for localized release to precisely modulate the tumor microenvironment, concurrently preventing tumor relapse and facilitating bone repair. However, challenges remain: hydrogel scaffolds typically lack sufficient mechanical strength for load-bearing applications and are, thus, most suitable for nonload-bearing defects. Future efforts may combine such systems with tissue-engineering scaffolds to improve their mechanical properties and clinical indications.

### Multimodal analytics to guide the selection and combination of therapeutic strategies: a future direction for immuno-tissue engineering in osteosarcoma

#### Multimodal analysis for optimizing immunotherapy in osteosarcoma

Increasing evidence indicates that the complexity and heterogeneity of the osteosarcoma tumor microenvironment (OS-TME) cannot be adequately captured by single-modality analytical approaches. In this context, multimodal analysis—integrating single-cell RNA sequencing, spatial transcriptomics, proteomics and radiomics—provides a more comprehensive framework for characterizing OS-TME dynamics by simultaneously incorporating transcriptomic features, protein expression profiles and imaging-derived metrics. This integrative strategy enables a more refined understanding of immune heterogeneity, spatial organization and functional states within the TME, particularly with respect to key immune populations such as tumor-associated macrophages with distinct polarization states, exhausted CD8^+^ T cells, Tregs and MDSCs [50]. For instance, spatial transcriptomic approaches delineate localized immune cell distribution patterns, whereas proteomic analyses quantify surface markers and cytokine profiles, thereby facilitating the identification of immune escape-associated mechanisms [166].

Beyond static characterization, multimodal analysis allows longitudinal assessment of the spatiotemporal evolution of the OS-TME during disease progression and therapeutic intervention. By combining single-cell sequencing with spatial transcriptomics, dynamic changes in immunosuppressive mediators, including PD-L1, CTLA-4, can be evaluated alongside fluctuations in pro-osteogenic signaling pathways involving BMP2, VEGF and TGF-β. Such analyses support the identification of actionable therapeutic targets and provide a data-driven basis for predicting treatment responses [51]. In clinical-relevant settings characterized by elevated PD-1 expression, combined blockade of PD-1 (e.g. nivolumab) and CSF-1R (e.g. PLX3397) has been shown to reshape macrophage polarization toward an antitumor phenotype, enhance CD8^+^ T-cell-mediated cytotoxicity and improve tumor control. Importantly, continuous monitoring of immune states helps mitigate treatment-associated risks, such as excessive inflammatory activation or recurrence-related signaling pathways [55, 117]. Complementary proteomic profiling further captures downstream immunological shifts, including changes in cytokine signatures, thereby facilitating coordinated rather than conflicting interactions between immunotherapy and tissue engineering-based repair strategies.

#### Guiding biomaterial design and drug delivery for coordinated immunomodulation and bone repair

In addition to guiding immunotherapeutic decision-making, multimodal analysis offers valuable insights for the rational design of biomaterials and drug delivery systems tailored to the spatiotemporal characteristics of osteosarcoma. By integrating multi-omics data with three-dimensional printing technologies, patient-specific scaffolds can be engineered with architectures and material compositions that better align with the heterogeneous distribution of immune and osteogenic cues within the OS-TME [167, 168]. Three-dimensionally printed scaffolds composed of hydroxyapatite, β-tricalcium phosphate or metallic materials such as titanium and tantalum can be fabricated using computer-aided design and manufacturing techniques to closely match defect geometries while providing hierarchical porosity, favorable biocompatibility and osteoinductive properties. The incorporation of functional agents—including photothermal or magnetothermal components, chemotherapeutic drugs or immunomodulatory molecules—further extends scaffold functionality toward simultaneous tumor control and bone regeneration [169–171].

Moreover, the integration of multimodal radiomics with artificial intelligence enables real-time assessment of therapeutic outcomes, including tumor burden, immune cell infiltration and bone density restoration, while machine-learning algorithms assist in anticipating resistance-associated trajectories. Upon detection of immunosuppressive cell accumulation, such as increased MDSCs, the adaptive incorporation of targeted inhibitors (e.g. STAT3 inhibitors like WP1066) into nanoscale delivery platforms may enhance antitumor efficacy without compromising normal bone remodeling processes [67].

Collectively, multimodal analysis provides a coherent framework for dissecting OS-TME heterogeneity and capturing its spatiotemporal dynamics. When integrated with the intrinsic properties of tissue engineering materials, this approach supports the development of immuno-tissue engineering strategies that are both mechanistically informed and clinically adaptable. Although this paradigm remains at an early stage of translational implementation, it offers new perspectives and opportunities for osteosarcoma treatment.

## Conclusions

Effective osteosarcoma management must simultaneously achieve tumor control and repair postoperative bone defects; however, the complexity and immunosuppressive nature of the OS-TME remain major obstacles. By dissecting the functions of immune cell populations within the OS-TME (e.g. TAMs, Tregs and MDSCs) and immune checkpoints (PD-1, CTLA-4 and CD47), this review highlights their central roles in immune evasion and bone regeneration. Strategies integrating immunotherapy with tissue engineering—such as temporally staged release, multimaterial hybrids, spatiotemporally responsive biomaterials and multimodal analytics-guided precision therapies—demonstrate the potential to suppress tumor recurrence while promoting bone repair. Patient-specific regimens developed through the integration of multiomics data and intelligent material design may further optimize efficacy, reduce recurrence risk and improve survival and quality of life in patients with osteosarcoma. Continued basic and clinical research will be essential to translate these approaches into practice.

## References

[1] Ritter J , Bielack SS. Osteosarcoma. Ann Oncol 2010;21:vii320–5. 20943636 10.1093/annonc/mdq276

[2] Xu M , Wang Z, Yu XC, Lin JH, Hu YC. Guideline for limb-salvage treatment of osteosarcoma. Orthop Surg 2020;12:1021–9. 32633103 10.1111/os.12702 PMC7454155

[3] Zhang B , Zhang Y, Li R, Li J, Lu X, Zhang Y. The efficacy and safety comparison of first-line chemotherapeutic agents (high-dose methotrexate, doxorubicin, cisplatin, and ifosfamide) for osteosarcoma: a network meta-analysis. J Orthop Surg Res 2020;15:51. 32054494 10.1186/s13018-020-1576-0 PMC7020590

[4] Horkoff MJ , Kendal JK, Blackmore C, Truong TH, Guilcher GMT, Brindle ME. A population-based analysis of the presentation and outcomes of pediatric patients with osteosarcoma in Canada: a report from CYP-C. Can J Surg 2022;65:e527–33. 35961660 10.1503/cjs.008220 PMC9377546

[5] Gaspar N , Occean BV, Pacquement H, Bompas E, Bouvier C, Brisse HJ, Castex MP, Cheurfa N, Corradini N, Delaye J, Entz-Werlé N, Gentet JC, Italiano A, Lervat C, Marec-Berard P, Mascard E, Redini F, Saumet L, Schmitt C, Tabone MD, Verite-Goulard C, Le Deley MC, Piperno-Neumann S, Brugieres L; UNICANCER Sarcoma Group. Results of methotrexate-etoposide-ifosfamide based regimen (M-EI) in osteosarcoma patients included in the French OS2006/sarcome-09 study. Eur J Cancer 2018;88:57–66. 29190507 10.1016/j.ejca.2017.09.036

[6] Grimer RJ. Surgical options for children with osteosarcoma. Lancet Oncol 2005;6:85–92. 15683817 10.1016/S1470-2045(05)01734-1

[7] Zraik IM , Heß-Busch Y. Management of chemotherapy side effects and their long-term sequelae. Urologe A 2021;60:862–71. 34185118 10.1007/s00120-021-01569-7

[8] Ho-Shui-Ling A , Bolander J, Rustom LE, Johnson AW, Luyten FP, Picart C. Bone regeneration strategies: engineered scaffolds, bioactive molecules and stem cells current stage and future perspectives. Biomaterials 2018;180:143–62. 30036727 10.1016/j.biomaterials.2018.07.017 PMC6710094

[9] Doppelt-Flikshtain O , Younis A, Tamari T, Ginesin O, Shentzer-Kutiel T, Nikomarov D, Bar-Sela G, Coyac BR, Assaraf YG, Zigdon-Giladi H. Endothelial progenitor cells promote osteosarcoma progression and invasiveness via AKT/PI3K signaling. Cancers (Basel) 2023;15:1818. 36980704 10.3390/cancers15061818 PMC10046883

[10] Chen H , Li C, Zhou T, Li X, Duarte MEL, Daubs MD, Buser Z, Brochmann EJ, Wang JC, Murray SS, Jiao L, Tian H. Secreted phosphoprotein 24 kD (Spp24) inhibits the growth of human osteosarcoma through the BMP-2/Smad signaling pathway. J Orthop Res 2023;41:1803–14. 36883270 10.1002/jor.25517

[11] Qi J , Liu S, Wu B, Xue G. The METTL3/TGF-β1 signaling axis promotes osteosarcoma progression by inducing MSC differentiation into CAFs via m(6)a modification. J Bone Oncol 2025;51:100662. 40034683 10.1016/j.jbo.2025.100662 PMC11875831

[12] Qi Y , Zhao M, Hu Y, Wang Y, Li P, Cao J, Shi M, Tan J, Zhang M, Xiao X, Xia J, Ma S, Qiao J, Yan Z, Li H, Pan B, Sang W, Li D, Li Z, Zhou J, Huang H, Liang A, Zheng J, Xu K. Efficacy and safety of CD19-specific CAR T cell-based therapy in B-cell acute lymphoblastic leukemia patients with CNSL. Blood 2022;139:3376–86. 35338773 10.1182/blood.2021013733 PMC11022988

[13] Grosser R , Cherkassky L, Chintala N, Adusumilli PS. Combination immunotherapy with CAR T cells and checkpoint blockade for the treatment of solid tumors. Cancer Cell 2019;36:471–82. 31715131 10.1016/j.ccell.2019.09.006 PMC7171534

[14] Yu S , Yao X. Advances on immunotherapy for osteosarcoma. Mol Cancer 2024;23:192. 39245737 10.1186/s12943-024-02105-9 PMC11382402

[15] Luo ZW , Liu PP, Wang ZX, Chen CY, Xie H. Macrophages in osteosarcoma immune microenvironment: implications for immunotherapy. Front Oncol 2020;10:586580. 33363016 10.3389/fonc.2020.586580 PMC7758531

[16] Anand N , Peh KH, Kolesar JM. Macrophage repolarization as a therapeutic strategy for osteosarcoma. Int J Mol Sci 2023;24:2858. 36769180 10.3390/ijms24032858 PMC9917837

[17] Bejarano L , Jordāo MJC, Joyce JA. Therapeutic targeting of the tumor microenvironment. Cancer Discov 2021;11:933–59. 33811125 10.1158/2159-8290.CD-20-1808

[18] Junttila MR , de Sauvage FJ. Influence of tumour micro-environment heterogeneity on therapeutic response. Nature 2013;501:346–54. 24048067 10.1038/nature12626

[19] Liang H , Cui M, Tu J, Chen X. Advancements in osteosarcoma management: integrating immune microenvironment insights with immunotherapeutic strategies. Front Cell Dev Biol 2024;12:1394339. 38915446 10.3389/fcell.2024.1394339 PMC11194413

[20] Quail DF , Joyce JA. Microenvironmental regulation of tumor progression and metastasis. Nat Med 2013;19:1423–37. 24202395 10.1038/nm.3394 PMC3954707

[21] Wu CC , Beird HC, Andrew Livingston J, Advani S, Mitra A, Cao S, Reuben A, Ingram D, Wang WL, Ju Z, Hong Leung C, Lin H, Zheng Y, Roszik J, Wang W, Patel S, Benjamin RS, Somaiah N, Conley AP, Mills GB, Hwu P, Gorlick R, Lazar A, Daw NC, Lewis V, Futreal PA. Immuno-genomic landscape of osteosarcoma. Nat Commun 2020;11:1008. 32081846 10.1038/s41467-020-14646-w PMC7035358

[22] Casanova JM , Almeida JS, Reith JD, Sousa LM, Fonseca R, Freitas-Tavares P, Santos-Rosa M, Rodrigues-Santos P. Tumor-infiltrating lymphocytes and cancer markers in osteosarcoma: influence on patient survival. Cancers (Basel) 2021;13:6075. 34885185 10.3390/cancers13236075 PMC8656728

[23] Fridman WH , Pagès F, Sautès-Fridman C, Galon J. The immune contexture in human tumours: impact on clinical outcome. Nat Rev Cancer 2012;12:298–306. 22419253 10.1038/nrc3245

[24] Hui E , Cheung J, Zhu J, Su X, Taylor MJ, Wallweber HA, Sasmal DK, Huang J, Kim JM, Mellman I, Vale RD. T cell costimulatory receptor CD28 is a primary target for PD-1-mediated inhibition. Science 2017;355:1428–33. 28280247 10.1126/science.aaf1292 PMC6286077

[25] Kim GR , Choi JM. Current understanding of cytotoxic T lymphocyte antigen-4 (CTLA-4) signaling in T-cell biology and disease therapy. Mol Cells 2022;45:513–21. 35950451 10.14348/molcells.2022.2056 PMC9385567

[26] Matlung HL , Szilagyi K, Barclay NA, van den Berg TK. The CD47-SIRPα signaling axis as an innate immune checkpoint in cancer. Immunol Rev 2017;276:145–64. 28258703 10.1111/imr.12527

[27] Jiang S , Li H, Zhang L, Mu W, Zhang Y, Chen T, Wu J, Tang H, Zheng S, Liu Y, Wu Y, Luo X, Xie Y, Ren J. Generic diagramming platform (GDP): a comprehensive database of high-quality biomedical graphics. Nucleic Acids Res 2025;53:D1670–76. 39470721 10.1093/nar/gkae973 PMC11701665

[28] Waldman AD , Fritz JM, Lenardo MJ. A guide to cancer immunotherapy: from T cell basic science to clinical practice. Nat Rev Immunol 2020;20:651–68. 32433532 10.1038/s41577-020-0306-5 PMC7238960

[29] Park JA , Cheung NV. Promise and challenges of T cell immunotherapy for osteosarcoma. Int J Mol Sci 2023;24:12520. 37569894 10.3390/ijms241512520 PMC10419531

[30] Raffin C , Vo LT, Bluestone JA. T(reg) cell-based therapies: challenges and perspectives. Nat Rev Immunol 2020;20:158–72. 31811270 10.1038/s41577-019-0232-6 PMC7814338

[31] Togashi Y , Shitara K, Nishikawa H. Regulatory T cells in cancer immunosuppression - implications for anticancer therapy. Nat Rev Clin Oncol 2019;16:356–71. 30705439 10.1038/s41571-019-0175-7

[32] Jhunjhunwala S , Hammer C, Delamarre L. Antigen presentation in cancer: insights into tumour immunogenicity and immune evasion. Nat Rev Cancer 2021;21:298–312. 33750922 10.1038/s41568-021-00339-z

[33] Pan Y , Yu Y, Wang X, Zhang T. Tumor-associated macrophages in tumor immunity. Front Immunol 2020;11:583084. 33365025 10.3389/fimmu.2020.583084 PMC7751482

[34] Mantovani A , Allavena P, Marchesi F, Garlanda C. Macrophages as tools and targets in cancer therapy. Nat Rev Drug Discov 2022;21:799–820. 35974096 10.1038/s41573-022-00520-5 PMC9380983

[35] Zhang W , Wang M, Ji C, Liu X, Gu B, Dong T. Macrophage polarization in the tumor microenvironment: emerging roles and therapeutic potentials. Biomed Pharmacother 2024;177:116930. 38878638 10.1016/j.biopha.2024.116930

[36] Hao J , Zhao X, Wang C, Cao X, Liu Y. Recent advances in nanoimmunotherapy by modulating tumor-associated macrophages for cancer therapy. Bioconjug Chem 2024;35:867–82. 38919067 10.1021/acs.bioconjchem.4c00242

[37] Liu W , Li L, Bai X, Zhang M, Lv W, Ma Y, Sun Y, Zhang H, Jiang Q, Yao Q, Zhang ZY. Osteosarcoma cell-derived migrasomes promote macrophage M2 polarization to aggravate osteosarcoma proliferation and metastasis. Adv Sci (Weinh) 2025;12:e2409870. 40056029 10.1002/advs.202409870 PMC12061288

[38] Qian BZ , Pollard JW. Macrophage diversity enhances tumor progression and metastasis. Cell 2010;141:39–51. 20371344 10.1016/j.cell.2010.03.014 PMC4994190

[39] Bai R , Li Y, Jian L, Yang Y, Zhao L, Wei M. The hypoxia-driven crosstalk between tumor and tumor-associated macrophages: mechanisms and clinical treatment strategies. Mol Cancer 2022;21:177. 36071472 10.1186/s12943-022-01645-2 PMC9454207

[40] Shaw P , Dwivedi SKD, Bhattacharya R, Mukherjee P, Rao G. VEGF signaling: role in angiogenesis and beyond. Biochim Biophys Acta Rev Cancer 2024;1879:189079. 38280470 10.1016/j.bbcan.2024.189079 PMC12927493

[41] Patel SS , Weirather JL, Lipschitz M, Lako A, Chen PH, Griffin GK, Armand P, Shipp MA, Rodig SJ. The microenvironmental niche in classic Hodgkin lymphoma is enriched for CTLA-4-positive T cells that are PD-1-negative. Blood 2019;134:2059–69. 31697809 10.1182/blood.2019002206 PMC7218752

[42] Feng S , Cheng X, Zhang L, Lu X, Chaudhary S, Teng R, Frederickson C, Champion MM, Zhao R, Cheng L, Gong Y, Deng H, Lu X. Myeloid-derived suppressor cells inhibit T cell activation through nitrating LCK in mouse cancers. Proc Natl Acad Sci USA 2018;115:10094–9. 30232256 10.1073/pnas.1800695115 PMC6176562

[43] Sholevar CJ , Liu NM, Mukarrama T, Kim J, Lawrence J, Canter RJ. Myeloid cells in the immunosuppressive microenvironment as immunotargets in osteosarcoma. Immunotargets Ther 2025;14:247–58. 40125425 10.2147/ITT.S485672 PMC11930235

[44] Li K , Shi H, Zhang B, Ou X, Ma Q, Chen Y, Shu P, Li D, Wang Y. Myeloid-derived suppressor cells as immunosuppressive regulators and therapeutic targets in cancer. Signal Transduct Target Ther 2021;6:362. 34620838 10.1038/s41392-021-00670-9 PMC8497485

[45] Zabeti Touchaei A , Vahidi S. Unraveling the interplay of CD8 + T cells and microRNA signaling in cancer: implications for immune dysfunction and therapeutic approaches. J Transl Med 2024;22:1131. 39707465 10.1186/s12967-024-05963-5 PMC11662517

[46] Chen Y , Yu D, Qian H, Shi Y, Tao Z. CD8(+) T cell-based cancer immunotherapy. J Transl Med 2024;22:394. 38685033 10.1186/s12967-024-05134-6 PMC11057112

[47] Ma K , Xu Y, Cheng H, Tang K, Ma J, Huang B. T cell-based cancer immunotherapy: opportunities and challenges. Sci Bull (Beijing) 2025;70:1872–90. 40221316 10.1016/j.scib.2025.03.054

[48] Rausch L , Kallies A. Molecular mechanisms governing CD8 T cell differentiation and checkpoint inhibitor response in cancer. Annu Rev Immunol 2025;43:515–43. 40279308 10.1146/annurev-immunol-082223-044122

[49] Fan Q , Wang Y, Cheng J, Pan B, Zang X, Liu R, Deng Y. Single-cell RNA-seq reveals T cell exhaustion and immune response landscape in osteosarcoma. Front Immunol 2024;15:1362970. 38629071 10.3389/fimmu.2024.1362970 PMC11018946

[50] Zhang Y , Jiang S, Lv J, Feng W, Yu Y, Zhao H. Osteosarcoma immune microenvironment: cellular struggle and novel therapeutic insights. Front Immunol 2025;16:1584450. 40534850 10.3389/fimmu.2025.1584450 PMC12174160

[51] Cheng D , Zhang Z, Mi Z, Tao W, Liu D, Fu J, Fan H. Deciphering the heterogeneity and immunosuppressive function of regulatory T cells in osteosarcoma using single-cell RNA transcriptome. Comput Biol Med 2023;165:107417. 37669584 10.1016/j.compbiomed.2023.107417

[52] Hennessy M , Wahba A, Felix K, Cabrera M, Segura MG, Kundra V, Ravoori MK, Stewart J, Kleinerman ES, Jensen VB, Gopalakrishnan V, Pena R, Quach P, Kim G, Kivimäe S, Madakamutil L, Overwijk WW, Zalevsky J, Gordon N. Bempegaldesleukin (BEMPEG; NKTR-214) efficacy as a single agent and in combination with checkpoint-inhibitor therapy in mouse models of osteosarcoma. Int J Cancer 2021;148:1928–37. 33152115 10.1002/ijc.33382 PMC7984260

[53] He J , Niu J, Wang L, Zhang W, He X, Zhang X, Hu W, Tang Y, Yang H, Sun J, Cui W, Shi Q. An injectable hydrogel microsphere-integrated training court to inspire tumor-infiltrating T lymphocyte potential. Biomaterials 2024;306:122475. 38306733 10.1016/j.biomaterials.2024.122475

[54] Yoshida K , Okamoto M, Sasaki J, Kuroda C, Ishida H, Ueda K, Ideta H, Kamanaka T, Sobajima A, Takizawa T, Tanaka M, Aoki K, Uemura T, Kato H, Haniu H, Saito N. Anti-PD-1 antibody decreases tumour-infiltrating regulatory T cells. BMC Cancer 2020;20:25. 31914969 10.1186/s12885-019-6499-y PMC6950856

[55] Fujiwara T , Yakoub MA, Chandler A, Christ AB, Yang G, Ouerfelli O, Rajasekhar VK, Yoshida A, Kondo H, Hata T, Tazawa H, Dogan Y, Moore MAS, Fujiwara T, Ozaki T, Purdue E, Healey JH. CSF1/CSF1R signaling inhibitor pexidartinib (PLX3397) reprograms tumor-associated macrophages and stimulates T-cell infiltration in the sarcoma microenvironment. Mol Cancer Ther 2021;20:1388–99. 34088832 10.1158/1535-7163.MCT-20-0591 PMC9336538

[56] Majzner RG , Theruvath JL, Nellan A, Heitzeneder S, Cui Y, Mount CW, Rietberg SP, Linde MH, Xu P, Rota C, Sotillo E, Labanieh L, Lee DW, Orentas RJ, Dimitrov DS, Zhu Z, Croix BS, Delaidelli A, Sekunova A, Bonvini E, Mitra SS, Quezado MM, Majeti R, Monje M, Sorensen PHB, Maris JM, Mackall CL. CAR T cells targeting B7-H3, a pan-cancer antigen, demonstrate potent preclinical activity against pediatric solid tumors and brain tumors. Clin Cancer Res 2019;25:2560–74. 30655315 10.1158/1078-0432.CCR-18-0432 PMC8456711

[57] Lake JA , Woods E, Hoffmeyer E, Schaller KL, Cruz-Cruz J, Fernandez J, Tufa D, Kooiman B, Hall SC, Jones D, Hayashi M, Verneris MR. Directing B7-H3 chimeric antigen receptor T cell homing through IL-8 induces potent antitumor activity against pediatric sarcoma. J Immunother Cancer 2024;12:e009221. 39043604 10.1136/jitc-2024-009221 PMC11268054

[58] Hegde S , Leader AM, Merad M. MDSC: markers, development, states, and unaddressed complexity. Immunity 2021;54:875–84. 33979585 10.1016/j.immuni.2021.04.004 PMC8709560

[59] Condamine T , Mastio J, Gabrilovich DI. Transcriptional regulation of myeloid-derived suppressor cells. J Leukoc Biol 2015;98:913–22. 26337512 10.1189/jlb.4RI0515-204R PMC4661041

[60] Ling Z , Yang C, Tan J, Dou C, Chen Y. Beyond immunosuppressive effects: dual roles of myeloid-derived suppressor cells in bone-related diseases. Cell Mol Life Sci 2021;78:7161–83. 34635950 10.1007/s00018-021-03966-9 PMC11072300

[61] Welte T , Kim IS, Tian L, Gao X, Wang H, Li J, Holdman XB, Herschkowitz JI, Pond A, Xie G, Kurley S, Nguyen T, Liao L, Dobrolecki LE, Pang L, Mo Q, Edwards DP, Huang S, Xin L, Xu J, Li Y, Lewis MT, Wang T, Westbrook TF, Rosen JM, Zhang XH. Oncogenic mTOR signalling recruits myeloid-derived suppressor cells to promote tumour initiation. Nat Cell Biol 2016;18:632–44. 27183469 10.1038/ncb3355 PMC4884142

[62] Wang Z , Jiang J, Li Z, Zhang J, Wang H, Qin Z. A myeloid cell population induced by freund adjuvant suppresses T-cell-mediated antitumor immunity. J Immunother 2010;33:167–77. 20145547 10.1097/CJI.0b013e3181bed2ba

[63] He S , Zheng L, Qi C. Myeloid-derived suppressor cells (MDSCs) in the tumor microenvironment and their targeting in cancer therapy. Mol Cancer 2025;24:5. 39780248 10.1186/s12943-024-02208-3 PMC11707952

[64] Safarzadeh E , Orangi M, Mohammadi H, Babaie F, Baradaran B. Myeloid-derived suppressor cells: important contributors to tumor progression and metastasis. J Cell Physiol 2018;233:3024–36. 28661031 10.1002/jcp.26075

[65] Peng D , Tanikawa T, Li W, Zhao L, Vatan L, Szeliga W, Wan S, Wei S, Wang Y, Liu Y, Staroslawska E, Szubstarski F, Rolinski J, Grywalska E, Stanisławek A, Polkowski W, Kurylcio A, Kleer C, Chang AE, Wicha M, Sabel M, Zou W, Kryczek I. Myeloid-derived suppressor cells endow stem-like qualities to breast cancer cells through IL6/STAT3 and NO/NOTCH cross-talk signaling. Cancer Res 2016;76:3156–65. 27197152 10.1158/0008-5472.CAN-15-2528 PMC4891237

[66] Hu T , Sun W, Jin Y, Dong Y, Liu W, Sun Z, Xiang Y, Chen Y. The combination of apatinib and antigen-specific DC-induced T cells exert antitumor effects by potently improving the immune microenvironment of osteosarcoma. Heliyon 2024;10:e36016. 39224314 10.1016/j.heliyon.2024.e36016 PMC11367533

[67] Shrestha P , Shrestha R, Zhou Y, Zielinski R, Priebe W, Kleinerman ES. STAT3 inhibition in combination with CD47 blockade inhibits osteosarcoma lung metastasis. Front Immunol 2025;16:1608375. 40529368 10.3389/fimmu.2025.1608375 PMC12170595

[68] Zhang Q , Sioud M. Tumor-associated macrophage subsets: shaping polarization and targeting. Int J Mol Sci 2023;24:7493. 37108657 10.3390/ijms24087493 PMC10138703

[69] Hume DA , MacDonald KP. Therapeutic applications of macrophage colony-stimulating factor-1 (CSF-1) and antagonists of CSF-1 receptor (CSF-1R) signaling. Blood 2012;119:1810–20. 22186992 10.1182/blood-2011-09-379214

[70] Chang YH , Huang YL, Tsai HC, Chang AC, Ko CY, Fong YC, Tang CH. Chemokine ligand 2 promotes migration in osteosarcoma by regulating the miR-3659/MMP-3 axis. Biomedicines 2023;11:2768. 37893141 10.3390/biomedicines11102768 PMC10604484

[71] Khan F , Pang L, Dunterman M, Lesniak MS, Heimberger AB, Chen P. Macrophages and microglia in glioblastoma: heterogeneity, plasticity, and therapy. J Clin Invest 2023;133:e163446. 36594466 10.1172/JCI163446 PMC9797335

[72] Biswas SK , Mantovani A. Macrophage plasticity and interaction with lymphocyte subsets: cancer as a paradigm. Nat Immunol 2010;11:889–96. 20856220 10.1038/ni.1937

[73] Wang Q , Ma W. Revisiting TAM polarization: beyond M1- and M2-type TAM toward clinical precision in macrophage-targeted therapy. Exp Mol Pathol 2025;143:104982. 40664070 10.1016/j.yexmp.2025.104982

[74] Jin R , Neufeld L, McGaha TL. Linking macrophage metabolism to function in the tumor microenvironment. Nat Cancer 2025;6:239–52. 39962208 10.1038/s43018-025-00909-2

[75] Majeti R , Chao MP, Alizadeh AA, Pang WW, Jaiswal S, Gibbs KD Jr., van Rooijen N, Weissman IL. CD47 is an adverse prognostic factor and therapeutic antibody target on human acute myeloid leukemia stem cells. Cell 2009;138:286–99. 19632179 10.1016/j.cell.2009.05.045 PMC2726837

[76] Wang Z , Li B, Li S, Lin W, Wang Z, Wang S, Chen W, Shi W, Chen T, Zhou H, Yinwang E, Zhang W, Mou H, Chai X, Zhang J, Lu Z, Ye Z. Metabolic control of CD47 expression through LAT2-mediated amino acid uptake promotes tumor immune evasion. Nat Commun 2022;13:6308. 36274066 10.1038/s41467-022-34064-4 PMC9588779

[77] Zhu Z , Zhang X, Lin X, Wang Y, Han C, Wang S. Research advances and application progress on miRNAs in exosomes derived from M2 macrophage for tissue injury repairing. Int J Nanomedicine 2025;20:1543–60. 39925680 10.2147/IJN.S508781 PMC11806736

[78] Wynn TA , Vannella KM. Macrophages in tissue repair, regeneration, and fibrosis. Immunity 2016;44:450–62. 26982353 10.1016/j.immuni.2016.02.015 PMC4794754

[79] Pajarinen J , Lin T, Gibon E, Kohno Y, Maruyama M, Nathan K, Lu L, Yao Z, Goodman SB. Mesenchymal stem cell-macrophage crosstalk and bone healing. Biomaterials 2019;196:80–9. 29329642 10.1016/j.biomaterials.2017.12.025 PMC6028312

[80] Goswami S , Sahai E, Wyckoff JB, Cammer M, Cox D, Pixley FJ, Stanley ER, Segall JE, Condeelis JS. Macrophages promote the invasion of breast carcinoma cells via a colony-stimulating factor-1/epidermal growth factor paracrine loop. Cancer Res 2005;65:5278–83. 15958574 10.1158/0008-5472.CAN-04-1853

[81] Cheng Z , Wang L, Wu C, Huang L, Ruan Y, Xue W. Tumor-derived exosomes induced M2 macrophage polarization and promoted the metastasis of osteosarcoma cells through tim-3. Arch Med Res 2021;52:200–10. 33162186 10.1016/j.arcmed.2020.10.018

[82] Curran S , Murray GI. Matrix metalloproteinases in tumour invasion and metastasis. J Pathol 1999;189:300–8. 10547590 10.1002/(SICI)1096-9896(199911)189:3<300::AID-PATH456>3.0.CO;2-C

[83] Cui Q , Wang X, Zhang Y, Shen Y, Qian Y. Macrophage-derived MMP-9 and MMP-2 are closely related to the rupture of the fibrous capsule of hepatocellular carcinoma leading to tumor invasion. Biol Proced Online 2023;25:8. 36918768 10.1186/s12575-023-00196-0 PMC10012540

[84] Zhu Z , Zhang H, Chen B, Liu X, Zhang S, Zong Z, Gao M. PD-L1-mediated immunosuppression in glioblastoma is associated with the infiltration and M2-polarization of tumor-associated macrophages. Front Immunol 2020;11:588552. 33329573 10.3389/fimmu.2020.588552 PMC7734279

[85] Chen L , Han X. Anti-PD-1/PD-L1 therapy of human cancer: past, present, and future. J Clin Invest 2015;125:3384–91. 26325035 10.1172/JCI80011 PMC4588282

[86] Lin EY , Nguyen AV, Russell RG, Pollard JW. Colony-stimulating factor 1 promotes progression of mammary tumors to malignancy. J Exp Med 2001;193:727–40. 11257139 10.1084/jem.193.6.727 PMC2193412

[87] Kondo H , Tazawa H, Fujiwara T, Yoshida A, Kure M, Demiya K, Kanaya N, Hata T, Uotani K, Hasei J, Kunisada T, Kagawa S, Yoshioka Y, Ozaki T, Fujiwara T. Osteosarcoma cell-derived CCL2 facilitates lung metastasis via accumulation of tumor-associated macrophages. Cancer Immunol Immunother 2025;74:193. 40343498 10.1007/s00262-025-04051-x PMC12064505

[88] Regan DP , Chow L, Das S, Haines L, Palmer E, Kurihara JN, Coy JW, Mathias A, Thamm DH, Gustafson DL, Dow SW. Losartan blocks osteosarcoma-elicited monocyte recruitment, and combined with the kinase inhibitor toceranib, exerts significant clinical benefit in canine metastatic osteosarcoma. Clin Cancer Res 2022;28:662–76. 34580111 10.1158/1078-0432.CCR-21-2105 PMC8866227

[89] Regan DP , Coy JW, Chahal KK, Chow L, Kurihara JN, Guth AM, Kufareva I, Dow SW. The angiotensin receptor blocker losartan suppresses growth of pulmonary metastases via AT1R-independent inhibition of CCR2 signaling and monocyte recruitment. J Immunol 2019;202:3087–102. 30971441 10.4049/jimmunol.1800619 PMC6504574

[90] Zhou Q , Xian M, Xiang S, Xiang D, Shao X, Wang J, Cao J, Yang X, Yang B, Ying M, He Q. All-trans retinoic acid prevents osteosarcoma metastasis by inhibiting M2 polarization of tumor-associated macrophages. Cancer Immunol Res 2017;5:547–59. 28515123 10.1158/2326-6066.CIR-16-0259

[91] Huang X , Wang L, Guo H, Zhang W. Macrophage membrane-coated nanovesicles for dual-targeted drug delivery to inhibit tumor and induce macrophage polarization. Bioact Mater 2023;23:69–79. 36406251 10.1016/j.bioactmat.2022.09.027 PMC9650013

[92] Wang J , Jin J, Chen T, Zhou Q. Curcumol synergizes with cisplatin in osteosarcoma by inhibiting M2-like polarization of tumor-associated macrophages. Molecules 2022;27:4345. 35889217 10.3390/molecules27144345 PMC9318016

[93] Richert I , Berchard P, Abbes L, Novikov A, Chettab K, Vandermoeten A, Dumontet C, Karanian M, Kerzerho J, Caroff M, Blay JY, Dutour A. A TLR4 agonist induces osteosarcoma regression by inducing an antitumor immune response and reprogramming M2 macrophages to M1 macrophages. Cancers (Basel) 2023;15:4635. 37760603 10.3390/cancers15184635 PMC10526955

[94] Wu H , Ma T, He M, Xie W, Wang X, Lu L, Wang H, Cui Y. Cucurbitacin B modulates M2 macrophage differentiation and attenuates osteosarcoma progression via PI3K/AKT pathway. Phytother Res 2024;38:2215–33. 38411031 10.1002/ptr.8146

[95] Cheng M , Jiang Y, Wang Y, Wu Y, Zhu Y. Enhancing osteosarcoma therapy through aluminium hydroxide nanosheets-enabled macrophage modulation. Int J Pharm 2024;649:123640. 38043749 10.1016/j.ijpharm.2023.123640

[96] Dermani FK , Samadi P, Rahmani G, Kohlan AK, Najafi R. PD-1/PD-L1 immune checkpoint: potential target for cancer therapy. J Cell Physiol 2019;234:1313–25. 30191996 10.1002/jcp.27172

[97] Yi M , Niu M, Xu L, Luo S, Wu K. Regulation of PD-L1 expression in the tumor microenvironment. J Hematol Oncol 2021;14:10. 33413496 10.1186/s13045-020-01027-5 PMC7792099

[98] Solinas C , Aiello M, Rozali E, Lambertini M, Willard-Gallo K, Migliori E. Programmed cell death-ligand 2: a neglected but important target in the immune response to cancer? Transl Oncol 2020;13:100811. 32622310 10.1016/j.tranon.2020.100811 PMC7332529

[99] Yokosuka T , Takamatsu M, Kobayashi-Imanishi W, Hashimoto-Tane A, Azuma M, Saito T. Programmed cell death 1 forms negative costimulatory microclusters that directly inhibit T cell receptor signaling by recruiting phosphatase SHP2. J Exp Med 2012;209:1201–17. 22641383 10.1084/jem.20112741 PMC3371732

[100] Zhang X , Schwartz JC, Guo X, Bhatia S, Cao E, Lorenz M, Cammer M, Chen L, Zhang ZY, Edidin MA, Nathenson SG, Almo SC. Structural and functional analysis of the costimulatory receptor programmed death-1. Immunity 2004;20:337–47. 15030777 10.1016/s1074-7613(04)00051-2

[101] Marasco M , Berteotti A, Weyershaeuser J, Thorausch N, Sikorska J, Krausze J, Brandt HJ, Kirkpatrick J, Rios P, Schamel WW, Köhn M, Carlomagno T. Molecular mechanism of SHP2 activation by PD-1 stimulation. Sci Adv 2020;6:eaay4458. 32064351 10.1126/sciadv.aay4458 PMC6994217

[102] Riley JL. PD-1 signaling in primary T cells. Immunol Rev 2009;229:114–25. 19426218 10.1111/j.1600-065X.2009.00767.x PMC3424066

[103] Parry RV , Chemnitz JM, Frauwirth KA, Lanfranco AR, Braunstein I, Kobayashi SV, Linsley PS, Thompson CB, Riley JL. CTLA-4 and PD-1 receptors inhibit T-cell activation by distinct mechanisms. Mol Cell Biol 2005;25:9543–53. 16227604 10.1128/MCB.25.21.9543-9553.2005 PMC1265804

[104] Patsoukis N , Brown J, Petkova V, Liu F, Li L, Boussiotis VA. Selective effects of PD-1 on akt and ras pathways regulate molecular components of the cell cycle and inhibit T cell proliferation. Sci Signal 2012;5:ra46. 22740686 10.1126/scisignal.2002796 PMC5498435

[105] Lin X , Kang K, Chen P, Zeng Z, Li G, Xiong W, Yi M, Xiang B. Regulatory mechanisms of PD-1/PD-L1 in cancers. Mol Cancer 2024;23:108. 38762484 10.1186/s12943-024-02023-w PMC11102195

[106] Zamani MR , Aslani S, Salmaninejad A, Javan MR, Rezaei N. PD-1/PD-L and autoimmunity: a growing relationship. Cell Immunol 2016;310:27–41. 27660198 10.1016/j.cellimm.2016.09.009

[107] Zheng W , Xiao H, Liu H, Zhou Y. Expression of programmed death 1 is correlated with progression of osteosarcoma. APMIS 2015;123:102–7. 25257510 10.1111/apm.12311

[108] Egen JG , Kuhns MS, Allison JP. CTLA-4: new insights into its biological function and use in tumor immunotherapy. Nat Immunol 2002;3:611–8. 12087419 10.1038/ni0702-611

[109] Esensten JH , Helou YA, Chopra G, Weiss A, Bluestone JA. CD28 costimulation: from mechanism to therapy. Immunity 2016;44:973–88. 27192564 10.1016/j.immuni.2016.04.020 PMC4932896

[110] Qureshi OS , Zheng Y, Nakamura K, Attridge K, Manzotti C, Schmidt EM, Baker J, Jeffery LE, Kaur S, Briggs Z, Hou TZ, Futter CE, Anderson G, Walker LS, Sansom DM. Trans-endocytosis of CD80 and CD86: a molecular basis for the cell-extrinsic function of CTLA-4. Science 2011;332:600–3. 21474713 10.1126/science.1202947 PMC3198051

[111] Zong Y , Deng K, Chong WP. Regulation of treg cells by cytokine signaling and co-stimulatory molecules. Front Immunol 2024;15:1387975. 38807592 10.3389/fimmu.2024.1387975 PMC11131382

[112] Hingorani P , Maas ML, Gustafson MP, Dickman P, Adams RH, Watanabe M, Eshun F, Williams J, Seidel MJ, Dietz AB. Increased CTLA-4(+) T cells and an increased ratio of monocytes with loss of class II (CD14(+) HLA-DR(lo/neg)) found in aggressive pediatric sarcoma patients. J Immunother Cancer 2015;3:35. 26286851 10.1186/s40425-015-0082-0 PMC4539889

[113] Oldenborg PA , Gresham HD, Lindberg FP. CD47-signal regulatory protein alpha (SIRPalpha) regulates fcgamma and complement receptor-mediated phagocytosis. J Exp Med 2001;193:855–62. 11283158 10.1084/jem.193.7.855 PMC2193364

[114] Li Y , Zhou H, Liu P, Lv D, Shi Y, Tang B, Xu J, Zhong T, Xu W, Zhang J, Zhou J, Ying K, Zhao Y, Sun Y, Jiang Z, Cheng H, Zhang X, Ke Y. SHP2 deneddylation mediates tumor immunosuppression in colon cancer via the CD47/SIRPα axis. J Clin Invest 2023;133:e162870. 36626230 10.1172/JCI162870 PMC9927946

[115] Ko Y , Park SY, Park JW, Kim JH, Kang HG, Lee JA. CD47 in osteosarcoma: correlation with metastasis and macrophage-mediated phagocytosis. Cells 2024;13:1862. 39594611 10.3390/cells13221862 PMC11592588

[116] Lussier DM , O’Neill L, Nieves LM, McAfee MS, Holechek SA, Collins AW, Dickman P, Jacobsen J, Hingorani P, Blattman JN. Enhanced T-cell immunity to osteosarcoma through antibody blockade of PD-1/PD-L1 interactions. J Immunother 2015;38:96–106. 25751499 10.1097/CJI.0000000000000065 PMC6426450

[117] Zheng B , Ren T, Huang Y, Sun K, Wang S, Bao X, Liu K, Guo W. PD-1 axis expression in musculoskeletal tumors and antitumor effect of nivolumab in osteosarcoma model of humanized mouse. J Hematol Oncol 2018;11:16. 29409495 10.1186/s13045-018-0560-1 PMC5801803

[118] Dhupkar P , Gordon N, Stewart J, Kleinerman ES. Anti-PD-1 therapy redirects macrophages from an M2 to an M1 phenotype inducing regression of OS lung metastases. Cancer Med 2018;7:2654–64. 29733528 10.1002/cam4.1518 PMC6010882

[119] Davis KL , Fox E, Merchant MS, Reid JM, Kudgus RA, Liu X, Minard CG, Voss S, Berg SL, Weigel BJ, Mackall CL. Nivolumab in children and young adults with relapsed or refractory solid tumours or lymphoma (ADVL1412): a multicentre, open-label, single-arm, phase 1-2 trial. Lancet Oncol 2020;21:541–50. 32192573 10.1016/S1470-2045(20)30023-1 PMC7255545

[120] D’Angelo SP , Richards AL, Conley AP, Woo HJ, Dickson MA, Gounder M, Kelly C, Keohan ML, Movva S, Thornton K, Rosenbaum E, Chi P, Nacev B, Chan JE, Slotkin EK, Kiesler H, Adamson T, Ling L, Rao P, Patel S, Livingston JA, Singer S, Agaram NP, Antonescu CR, Koff A, Erinjeri JP, Hwang S, Qin LX, Donoghue MTA, Tap WD. Pilot study of bempegaldesleukin in combination with nivolumab in patients with metastatic sarcoma. Nat Commun 2022;13:3477. 35710741 10.1038/s41467-022-30874-8 PMC9203519

[121] Tang G , Zhang Q, Wang F, Zhang H, Qi Y. Combination of sintilimab and anlotinib for metastatic osteosarcoma: a case report. Onco Targets Ther 2024;17:661–5. 39161887 10.2147/OTT.S464678 PMC11331146

[122] Song G , Tang Q, Lu J, Xu H, Wang A, Deng C, Wu H, Hu J, Zhu X, Wang J. Lenvatinib monotherapy versus lenvatinib in combination with PD-1 blockades as Re-challenging treatment for patients with metastatic osteosarcoma: a real-world study. Drug Des Devel Ther 2025;19:1119–28. 10.2147/DDDT.S501742 PMC11846616 39991085

[123] Li J , Ding Z, Liu J, Li G, Li Y, Wang W, Nundlall K, Deng Y, Miao J, Hu M, Chen S, Zeng D, Cao L. Reshaping tumor immune microenvironment and modulating T cell function based on hierarchical nanotherapeutics for synergistically inhibiting osteosarcoma. Mater Today Bio 2025;34:102095. 10.1016/j.mtbio.2025.102095 PMC12320706 40761512

[124] Merchant MS , Wright M, Baird K, Wexler LH, Rodriguez-Galindo C, Bernstein D, Delbrook C, Lodish M, Bishop R, Wolchok JD, Streicher H, Mackall CL. Phase I clinical trial of ipilimumab in pediatric patients with advanced solid tumors. Clin Cancer Res 2016;22:1364–70. 26534966 10.1158/1078-0432.CCR-15-0491 PMC5027962

[125] Somaiah N , Conley AP, Parra ER, Lin H, Amini B, Solis Soto L, Salazar R, Barreto C, Chen H, Gite S, Haymaker C, Nassif EF, Bernatchez C, Mitra A, Livingston JA, Ravi V, Araujo DM, Benjamin R, Patel S, Zarzour MA, Sabir S, Lazar AJ, Wang WL, Daw NC, Zhou X, Roland CL, Cooper ZA, Rodriguez-Canales J, Futreal A, Soria JC, Wistuba II, Hwu P. Durvalumab plus tremelimumab in advanced or metastatic soft tissue and bone sarcomas: a single-centre phase 2 trial. Lancet Oncol 2022;23:1156–66. 35934010 10.1016/S1470-2045(22)00392-8

[126] Xu JF , Pan XH, Zhang SJ, Zhao C, Qiu BS, Gu HF, Hong JF, Cao L, Chen Y, Xia B, Bi Q, Wang YP. CD47 blockade inhibits tumor progression human osteosarcoma in xenograft models. Oncotarget 2015;6:23662–70. 26093091 10.18632/oncotarget.4282 PMC4695143

[127] Ramay HR , Zhang M. Preparation of porous hydroxyapatite scaffolds by combination of the gel-casting and polymer sponge methods. Biomaterials 2003;24:3293–302. 12763457 10.1016/s0142-9612(03)00171-6

[128] Bohner M , Santoni BLG, Döbelin N. β-tricalcium phosphate for bone substitution: synthesis and properties. Acta Biomater 2020;113:23–41. 32565369 10.1016/j.actbio.2020.06.022

[129] Zhang F , Yang J, Zuo Y, Li K, Mao Z, Jin X, Zhang S, Gao H, Cui Y. Digital light processing of β-tricalcium phosphate bioceramic scaffolds with controllable porous structures for patient specific craniomaxillofacial bone reconstruction. Mater Des 2022;216:110558.

[130] Bouler JM , Pilet P, Gauthier O, Verron E. Biphasic calcium phosphate ceramics for bone reconstruction: a review of biological response. Acta Biomater 2017;53:1–12. 28159720 10.1016/j.actbio.2017.01.076

[131] Lim HK , Hong SJ, Byeon SJ, Chung SM, On SW, Yang BE, Lee JH, Byun SH. 3D-printed ceramic bone scaffolds with variable pore architectures. Int J Mol Sci 2020;21:6942. 32971749 10.3390/ijms21186942 PMC7555666

[132] Ramay HR , Zhang M. Biphasic calcium phosphate nanocomposite porous scaffolds for load-bearing bone tissue engineering. Biomaterials 2004;25:5171–80. 15109841 10.1016/j.biomaterials.2003.12.023

[133] Chen F , Han J, Guo Z, Mu C, Yu C, Ji Z, Sun L, Wang Y, Wang J. Antibacterial 3D-printed silver nanoparticle/poly lactic-co-glycolic acid (PLGA) scaffolds for bone tissue engineering. Materials (Basel) 2023;16:3895. 37297029 10.3390/ma16113895 PMC10253518

[134] Mushtaq RT , Askari GH, Bao C, Wang Y, Ahmed K, Khan AM, Sharma S, Alkahtani M. Optimization of 3D-printed bio-based super tough PLA (ST-PLA) scaffolds for cancellous bone regeneration: mechanical properties, lattice architecture, and osseointegration potential. Int J Biol Macromol 2025;316:144466. 40403814 10.1016/j.ijbiomac.2025.144466

[135] Zhou A , Liao J, Huang Z, Zeng K, Guo Y, Hou X, Zhao H. A 3D-printed PLA honeycomb-shaped scaffolds for bone tissue engineering. J Biomater Appl 2025;8853282251396800. 10.1177/08853282251396800 41215570

[136] Jang JW , Min KE, Kim C, Wern C, Yi S. PCL and DMSO_2_ composites for bio-scaffold materials. Materials (Basel) 2023;16:2481. 36984361 10.3390/ma16062481 PMC10055993

[137] Sakarya D , Zorlu T, Yücel S, Sahin YM, Özarslan AC. Advanced bioresin formulation for 3D-printed bone scaffolds: PCLDMA and p-PLA integration. Polymers (Basel) 2024;16:534. 38399911 10.3390/polym16040534 PMC10892561

[138] Carraro F , Bagno A. Tantalum as trabecular metal for endosseous implantable applications. Biomimetics (Basel) 2023;8:49. 36810380 10.3390/biomimetics8010049 PMC9944482

[139] Jiang C , Gong G, Xiao S, Zhang S, Chen D, Song S, Dai H, Wu C, Zou Q, Li J, Wen B. Mechanical and biological properties of 3D-printed porous titanium scaffolds coated with composite growth factors. BMC Oral Health 2025;25:808. 40426159 10.1186/s12903-025-06110-2 PMC12107813

[140] Surendran AK , Jayaraj J, Veerappan R, Gupta M, Amirthalingam S, R KG. Gd added Mg alloy for biodegradable implant applications. J Biomed Mater Res B Appl Biomater 2024;112:e35474. 39215555 10.1002/jbm.b.35474

[141] Wang X , Liu A, Zhang Z, Hao D, Liang Y, Dai J, Jin X, Deng H, Zhao Y, Wen P, Li Y. Additively manufactured Zn-2Mg alloy porous scaffolds with customizable biodegradable performance and enhanced osteogenic ability. Adv Sci (Weinh) 2024;11:e2307329. 38059810 10.1002/advs.202307329 PMC10837348

[142] Ji H , Shen G, Liu H, Liu Y, Qian J, Wan G, Luo E. Biodegradable Zn-2Cu-0.5Zr alloy promotes the bone repair of senile osteoporotic fractures via the immune-modulation of macrophages. Bioact Mater 2024;38:422–37. 38770427 10.1016/j.bioactmat.2024.05.003 PMC11103781

[143] Qin Y , Liu A, Guo H, Shen Y, Wen P, Lin H, Xia D, Voshage M, Tian Y, Zheng Y. Additive manufacturing of Zn-Mg alloy porous scaffolds with enhanced osseointegration: in vitro and in vivo studies. Acta Biomater 2022;145:403–15. 35381400 10.1016/j.actbio.2022.03.055

[144] Ye J , Miao B, Xiong Y, Guan Y, Lu Y, Jia Z, Wu Y, Sun X, Guan C, He R, Xiong X, Jia H, Jiang H, Liu Z, Zhang Y, Wei Y, Lin W, Wang A, Wang Y, Meng H, Xu W, Yuan G, Peng J. 3D printed porous magnesium metal scaffolds with bioactive coating for bone defect repair: enhancing angiogenesis and osteogenesis. J Nanobiotechnology 2025;23:160. 40033312 10.1186/s12951-025-03222-3 PMC11874660

[145] Baino F , Dias J, Alidoost M, Schwentenwein M, Verné E. Making foam-like bioactive glass scaffolds by vat photopolymerization. Open Ceramics 2023;15:100392.

[146] Koushik TM , Miller CM, Antunes E. Bone tissue engineering scaffolds: function of multi-material hierarchically structured scaffolds. Adv Healthc Mater 2023;12:e2202766. 36512599 10.1002/adhm.202202766 PMC11468595

[147] Mobini S , Song YH, McCrary MW, Schmidt CE. Advances in ex vivo models and lab-on-a-chip devices for neural tissue engineering. Biomaterials 2019;198:146–66. 29880219 10.1016/j.biomaterials.2018.05.012 PMC6957334

[148] Maleki Dana P , Hallajzadeh J, Asemi Z, Mansournia MA, Yousefi B. Chitosan applications in studying and managing osteosarcoma. Int J Biol Macromol 2021;169:321–9. 33310094 10.1016/j.ijbiomac.2020.12.058

[149] Bozorgi A , Sabouri L. Osteosarcoma, personalized medicine, and tissue engineering; an overview of overlapping fields of research. Cancer Treat Res Commun 2021;27:100324. 33517237 10.1016/j.ctarc.2021.100324

[150] Yang Q , Yin H, Xu T, Zhu D, Yin J, Chen Y, Yu X, Gao J, Zhang C, Chen Y, Gao Y. Engineering 2D mesoporous silica@MXene-integrated 3D-printing scaffolds for combinatory osteosarcoma therapy and NO-augmented bone regeneration. Small 2020;16:e1906814. 32108432 10.1002/smll.201906814

[151] Joy K , David SS, Shanmugavadivu A, Selvamurugan N, Mani P. Three-dimensional porous polycaprolactone/chitosan/bioactive glass scaffold for bone tissue engineering. J Biomater Sci Polym Ed 2024;35:2829–44. 39185697 10.1080/09205063.2024.2391218

[152] Liu X , Xu J, Zhu J, Wang Z, Fang H, Guo W, Cao L, Cheng YY, Song K. Multimodal therapy process engineering mediated by 3D-printed scaffolds for enhanced postoperative osteosarcoma regeneration. Tissue Cell 2026;98:103212. 41187387 10.1016/j.tice.2025.103212

[153] Puri A , Gulia A, Pruthi M. Outcome of surgical resection of pelvic osteosarcoma. Indian J Orthop 2014;48:273–8. 24932033 10.4103/0019-5413.132515 PMC4052026

[154] Zhao J , Zhang ZR, Zhao N, Ma BA, Fan QY. VEGF silencing inhibits human osteosarcoma angiogenesis and promotes cell apoptosis via PI3K/AKT signaling pathway. Int J Clin Exp Med 2015;8:12411–7. 26550152 PMC4612837

[155] Tian H , Zhou T, Chen H, Li C, Jiang Z, Lao L, Kahn SA, Duarte MEL, Zhao J, Daubs MD, Buser Z, Brochmann EJ, Wang JC, Murray SS. Bone morphogenetic protein-2 promotes osteosarcoma growth by promoting epithelial-mesenchymal transition (EMT) through the wnt/β-catenin signaling pathway. J Orthop Res 2019;37:1638–48. 30737824 10.1002/jor.24244

[156] Bessa-Gonçalves M , Ribeiro-Machado C, Costa M, Ribeiro CC, Barbosa JN, Barbosa MA, Santos SG. Magnesium incorporation in fibrinogen scaffolds promotes macrophage polarization towards M2 phenotype. Acta Biomater 2023;155:667–83. 36328124 10.1016/j.actbio.2022.10.046

[157] Abedi N , Sadeghian A, Kouhi M, Haugen HJ, Savabi O, Nejatidanesh F. Immunomodulation in bone tissue engineering: recent advancements in scaffold design and biological modifications for enhanced regeneration. ACS Biomater Sci Eng 2025;11:1269–90. 39970366 10.1021/acsbiomaterials.4c01613

[158] Gou M , Wang H, Xie H, Song H. Macrophages in guided bone regeneration: potential roles and future directions. Front Immunol 2024;15:1396759. 38736888 10.3389/fimmu.2024.1396759 PMC11082316

[159] Zhang X , Zu H, Zhao D, Yang K, Tian S, Yu X, Lu F, Liu B, Yu X, Wang B, Wang W, Huang S, Wang Y, Wang Z, Zhang Z. Ion channel functional protein kinase TRPM7 regulates Mg ions to promote the osteoinduction of human osteoblast via PI3K pathway: in vitro simulation of the bone-repairing effect of Mg-based alloy implant. Acta Biomater 2017;63:369–82. 28882757 10.1016/j.actbio.2017.08.051

[160] Li C , Zhang W, Nie Y, Du X, Huang C, Li L, Long J, Wang X, Tong W, Qin L, Lai Y. Time-sequential and multi-functional 3D printed MgO(2)/PLGA scaffold developed as a novel biodegradable and bioactive bone substitute for challenging postsurgical osteosarcoma treatment. Adv Mater 2024;36:e2308875. 38091500 10.1002/adma.202308875

[161] Huang H , Qiang L, Fan M, Liu Y, Yang A, Chang D, Li J, Sun T, Wang Y, Guo R, Zhuang H, Li X, Guo T, Wang J, Tan H, Zheng P, Weng J. 3D-printed tri-element-doped hydroxyapatite/ polycaprolactone composite scaffolds with antibacterial potential for osteosarcoma therapy and bone regeneration. Bioact Mater 2024;31:18–37. 37593495 10.1016/j.bioactmat.2023.07.004 PMC10432151

[162] Lu Y , Liu A, Jin S, Dai J, Yu Y, Wen P, Zheng Y, Xia D. Additively manufactured biodegradable Zn-based porous scaffolds to suppress osteosarcoma and promote osteogenesis. Adv Mater 2025;37:e2410589. 39564691 10.1002/adma.202410589

[163] Bian Y , Zhao K, Hu T, Tan C, Liang R, Weng X. A Se nanoparticle/MgFe-LDH composite nanosheet as a multifunctional platform for osteosarcoma eradication, antibacterial and bone reconstruction. Adv Sci (Weinh) 2024;11:e2403791. 38958509 10.1002/advs.202403791 PMC11434235

[164] Zhang D , Cheng S, Tan J, Xie J, Zhang Y, Chen S, Du H, Qian S, Qiao Y, Peng F, Liu X. Black Mn-containing layered double hydroxide coated magnesium alloy for osteosarcoma therapy, bacteria killing, and bone regeneration. Bioact Mater 2022;17:394–405. 35386440 10.1016/j.bioactmat.2022.01.032 PMC8965036

[165] Chu X , Mi B, Xiong Y, Wang R, Liu T, Hu L, Yan C, Zeng R, Lin J, Fu H, Liu G, Zhang K, Bian L. Bioactive nanocomposite hydrogel enhances postoperative immunotherapy and bone reconstruction for osteosarcoma treatment. Biomaterials 2025;312:122714. 39079462 10.1016/j.biomaterials.2024.122714

[166] Zhou Y , Yang D, Yang Q, Lv X, Huang W, Zhou Z, Wang Y, Zhang Z, Yuan T, Ding X, Tang L, Zhang J, Yin J, Huang Y, Yu W, Wang Y, Zhou C, Su Y, He A, Sun Y, Shen Z, Qian B, Meng W, Fei J, Yao Y, Pan X, Chen P, Hu H. Single-cell RNA landscape of intratumoral heterogeneity and immunosuppressive microenvironment in advanced osteosarcoma. Nat Commun 2020;11:6322. 33303760 10.1038/s41467-020-20059-6 PMC7730477

[167] Liu X , Liu Y, Qiang L, Ren Y, Lin Y, Li H, Chen Q, Gao S, Yang X, Zhang C, Fan M, Zheng P, Li S, Wang J. Multifunctional 3D-printed bioceramic scaffolds: recent strategies for osteosarcoma treatment. J Tissue Eng 2023;14:20417314231170371. 37205149 10.1177/20417314231170371 PMC10186582

[168] Rong X , Xiao S, Geng W, Zhu B, Mou P, Ding Z, Zhang B, Fan Y, Qiu L, Cheng C. Sono-activable and biocatalytic 3D-printed scaffolds for intelligently sequential therapies in osteosarcoma eradication and defect regeneration. Nat Commun 2025;16:6150. 40610512 10.1038/s41467-025-61377-x PMC12229518

[169] Bai Y , Wu N, Li X, Liu Z, Li K, Jiao T, Liu F. Recent progress of 3D printed responsive scaffolds for bone repair: a review. Mater Today Bio 2025;35:102351. 10.1016/j.mtbio.2025.102351 PMC12510199 41080731

[170] Jian G , Wang S, Wang X, Lu Q, Zhu X, Wan S, Wang S, Li D, Wang C, He Q, Chen T, Song J. Enhanced sequential osteosarcoma therapy using a 3D-printed bioceramic scaffold combined with 2D nanosheets via NIR-II photothermal-chemodynamic synergy. Bioact Mater 2025;50:540–55. 40391104 10.1016/j.bioactmat.2025.04.029 PMC12088772

[171] Ma Y , Zhang B, Sun H, Liu D, Zhu Y, Zhu Q, Liu X. The dual effect of 3D-printed biological scaffolds composed of diverse biomaterials in the treatment of bone tumors. Int J Nanomedicine 2023;18:293–305. 36683596 10.2147/IJN.S390500 PMC9851059

